# EZH2 inhibition remodels the inflammatory senescence-associated secretory phenotype to potentiate pancreatic cancer immune surveillance

**DOI:** 10.1038/s43018-023-00553-8

**Published:** 2023-05-04

**Authors:** Loretah Chibaya, Katherine C. Murphy, Kelly D. DeMarco, Sneha Gopalan, Haibo Liu, Chaitanya N. Parikh, Yvette Lopez-Diaz, Melissa Faulkner, Junhui Li, John P. Morris, Yu-jui Ho, Sachliv K. Chana, Janelle Simon, Wei Luan, Amanda Kulick, Elisa de Stanchina, Karl Simin, Lihua Julie Zhu, Thomas G. Fazzio, Scott W. Lowe, Marcus Ruscetti

**Affiliations:** 1 Department of Molecular, Cell, and Cancer Biology, University of Massachusetts Chan Medical School, Worcester, MA, USA; 2 Program in Molecular Medicine, University of Massachusetts Chan Medical School, Worcester, MA, USA; 3 Department of Cancer Biology and Genetics, Memorial Sloan Kettering Cancer Center, New York, NY, USA; 4 Department of Molecular Pharmacology, Memorial Sloan Kettering Cancer Center, New York, NY, USA; 5 Program in Bioinformatics and Integrative Biology, University of Massachusetts Chan Medical School, Worcester, MA, USA; 6 Howard Hughes Medical Institute, Chevy Chase, MD 20815, USA; 7 Immunology and Microbiology Program, University of Massachusetts Medical Chan School, Worcester, MA, USA; 8 Cancer Center, University of Massachusetts Medical Chan School, Worcester, MA. USA

## Abstract

Immunotherapies that produce durable responses in some malignancies have failed in pancreatic ductal adenocarcinoma (PDAC) due to rampant immune suppression and poor tumor immunogenicity. We and others have demonstrated that induction of the senescence-associated secretory phenotype (SASP) can be an effective approach to activate anti-tumor Natural Killer (NK) and T cell immunity. Here we found the pancreas tumor microenvironment (TME) suppresses NK and T cell surveillance following therapy-induced senescence through EZH2-mediated epigenetic repression of pro-inflammatory SASP genes. EZH2 blockade stimulated production of SASP chemokines CCL2 and CXCL9/10, leading to enhanced NK and T cell infiltration and PDAC eradication in mouse models. EZH2 activity was also associated with suppression of chemokine signaling and cytotoxic lymphocytes and reduced survival in PDAC patients. These results demonstrate that EZH2 represses of the pro-inflammatory SASP, and that EZH2 inhibition combined with senescence-inducing therapy could be a powerful means to achieve immune-mediated tumor control in PDAC.

Pancreatic ductal adenocarcinoma (PDAC) is a devastating disease with few effective treatment options, and has recently risen to become the 3^rd^ leading cause of cancer-related death^1^. Conventional chemotherapy regimens have limited efficacy in PDAC, in part due to a fibrotic and desmoplastic tumor microenvironment (TME) that leads to vascular dysfunction and poor drug delivery and activity in tumors^2–4^. Immunotherapy regimens including chimeric antigen receptor (CAR) T cells and anti-PD-1 and CTLA-4 immune checkpoint blockade (ICB) therapies that have been effective in other aggressive, chemo-refractory tumors have been ineffective in PDAC because of widespread innate and adaptive immune suppression in the pancreas TME^5–7^. Indeed, an abundance of suppressive macrophage and myeloid populations, poor tumor immunogenicity, and a lack of cytotoxic Natural Killer (NK) and T cell infiltration contribute to the immunologically “cold” TME associated with PDAC and immunotherapy resistance^8^. Thus, new and innovative approaches are needed to target the multiple axes of immune suppression in PDAC to achieve durable therapeutic outcomes.

Point mutations in KRAS are oncogenic drivers in PDAC and found in >90% of patients^9^. One strategy to increase tumor immunogenicity and stimulate anti-tumor immunity is to target the oncogenic pathways that drive immune suppression^10,11^, including RAS signaling itself^12,13^. RAS pathway targeting therapies have been shown not only to increase tumor immunogenicity through upregulating antigen presentation and processing genes (e.g. major histocompatibility complex (MHC) Class I (MHC-I) molecules) but also lead to immune stimulatory microenvironments that activate anti-tumor NK and T cell immunity and ICB therapy efficacy^14–18^. We previously showed that the combination of the MEK inhibitor trametinib and CDK4/6 inhibitor palbociclib (T/P) could trigger KRAS mutant cancers to enter cellular senescence, a stable cell cycle arrest program that is accompanied by a secretory program that can modulate immune responses^19,20^. This senescence-associated secretory phenotype (SASP) includes a collection of pleiotropic factors such as pro- and anti-inflammatory chemokines and cytokines, angiogenic factors, growth and stemness components, matrix metalloproteinases (MMPs), and lipid species that remodel the surrounding TME in both tumor promoting and tumor suppressive ways depending on the context^21–23^.

In certain tumor and cancer therapy contexts, we and others have demonstrated that the SASP can mediate potent anti-tumor immunity to block tumor formation, regress established tumors, and enhance immunotherapy regimens^19,20,24–27^. Recently we found that therapy-induced senescence following T/P treatment could induce anti-tumor immune surveillance in preclinical mouse models of KRAS mutant lung adenocarcinoma (LUAD) and PDAC. In KRAS mutant LUAD, T/P-induced senescence led to secretion of pro-inflammatory SASP factors that activated NK cell immune surveillance and drove NK cell-mediated long-term lung tumor responses^19^. Intriguingly, in similar genetic models of KRAS mutant PDAC, T/P treatment led to a predominantly pro-angiogenic SASP that enhanced vascularization and CD8^+^ T cell extravasation into PDAC with little effect on NK cell immunity^20^. Combining therapy-induced senescence with anti-PD-1 ICB enhanced cytotoxic T cell immunity and led to tumor responses in PDAC-bearing animals, demonstrating that the SASP could be a means to make “cold” PDAC tumors “hot” and potentiate currently ineffective ICB strategies.

In order to effectively harness senescence and its immune stimulating properties for tumor suppression, we need a better understanding of why the SASP elicits altered immune responses in different cancer contexts and how the SASP transcriptional program or specific SASP factors can be optimized for immune-mediated tumor destruction^28^. In the setting of PDAC, it will be important to elucidate how the pancreas TME suppresses cytotoxic NK cells that can act as potent eliminators of senescent tumor cells^29^. As NK cells can both directly eradicate target cells through release of cytolytic granules, as well as indirectly mobilize adaptive T cell immunity through secretion of cytokines and chemokines, they are promising targets for cancer immunotherapy^30^. Here we set out to address why the SASP elicited different immune responses in the pancreas and how it could be harnessed for NK cell immunotherapy in PDAC.

## RESULTS

### Senescence induces NK cell immunity in the lung but not pancreas TME

**Extended Data Fig. 1.**
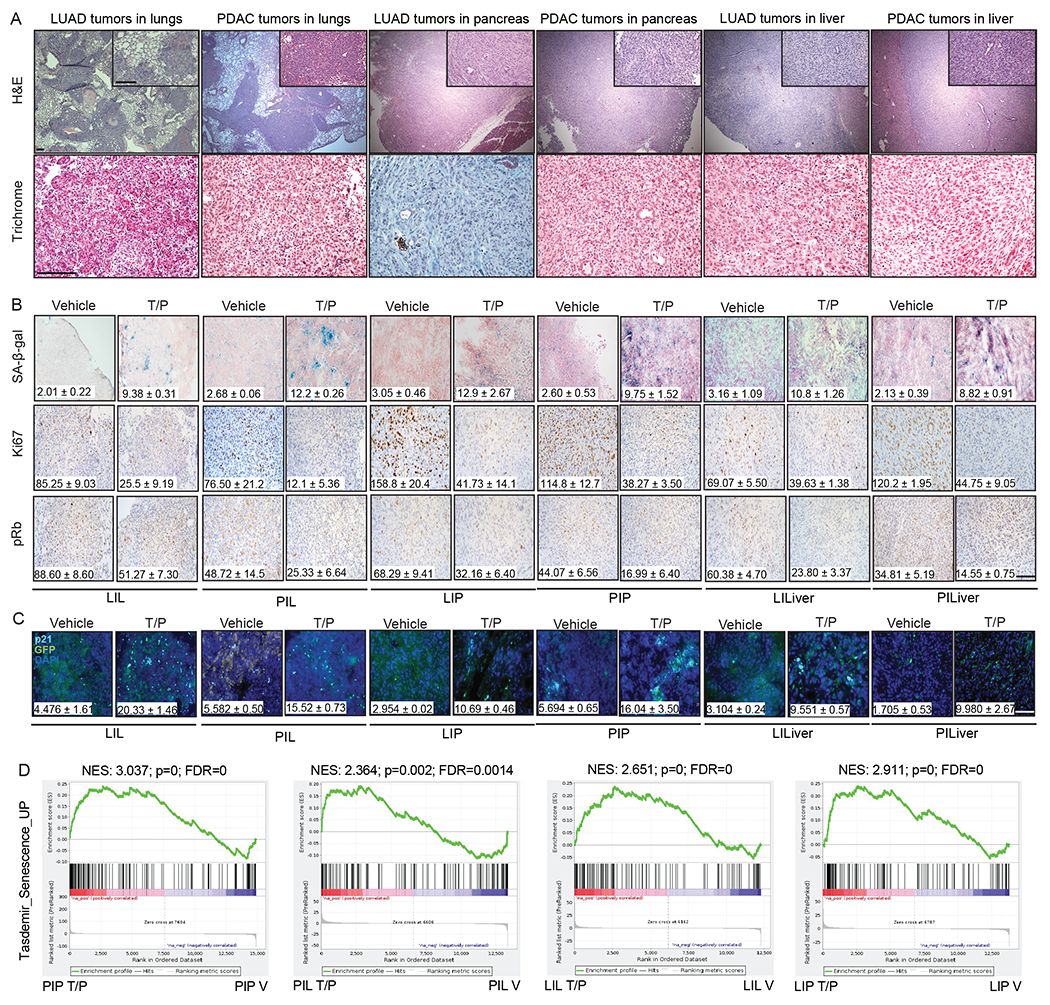
T/P treatment induces cellular senescence across tumor conditions *in vivo*. **a**, Representative Haematoxylin and eosin (H&E) (top) and Masson’s trichrome (bottom) staining of indicated *KPC1* PDAC (PIP, PIL, PILiver) and *KP1* LUAD (LIL, LIP, LILiver) derived-tumors from 2-3 independent experiments. Scale bars, 100μm. **b**, Immunohistochemical (IHC) staining of indicated *KPC1* PDAC (PIP, PIL, PILiver) and *KP1* LUAD (LIL, LIP, LILiver) derived-tumors treated with vehicle (V) or combined trametinib (1mg/kg) and palbociclib (100 mg/kg) (T/P) for 2 weeks. Quantification of the percentage of SA-β-gal^+^ area and the number of Ki67^+^ and pRb^+^ cells per field are shown inset (n=2-4 per group). Scale bar, 50μm. **c,** Immunofluorescence staining of indicated *KPC1* PDAC (PIP, PIL, PILiver) and *KP1* LUAD (LIL, LIP, LILiver) derived-tumors grown in different organs and treated as in (**b**). Quantification of the percentage of GFP^+^ (green) tumor cells expressing p21 (cyan) is shown inset (n=2-4 per group). Scale bar, 50μm. **d**, GFP^+^ tumor cells were FACS sorted from indicated tumors and extracted RNA subjected to RNA-seq analysis (n=2-4 per group). Gene Set Enrichment Analysis (GSEA) of RNA-seq data using an established senescence gene set is shown. NES, normalized enrichment score. *P* values in **d** were calculated using two-sided, Kolmogorov-Smirnov test. Error bars, mean ± SEM.

**Fig. 1.**
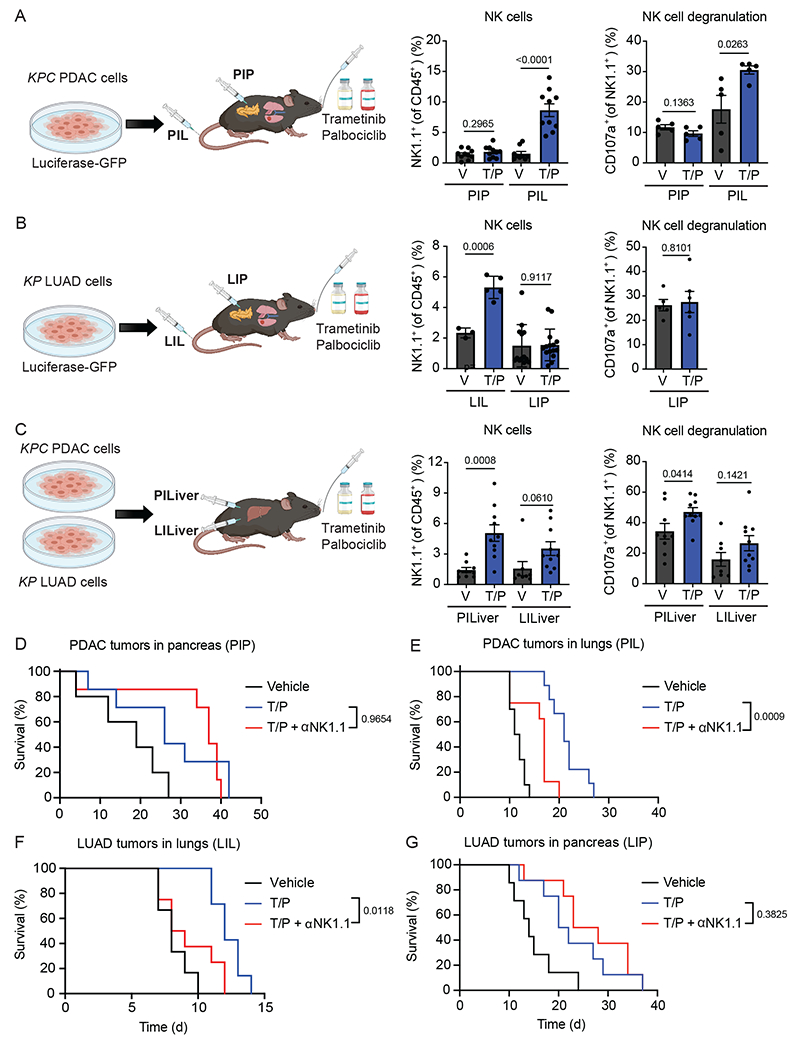
NK cell immunity is induced in the lung but not pancreas TME following therapy-induced senescence. **a-b**, *KPC1* PDAC (**a**) or *KP1* LUAD (**b**) tumor cells expressing luciferase-GFP were injected i.v. or orthotopically into the pancreas of 8-12 week old C57BL/6 female mice. Following tumor formation in the lungs or pancreas, mice were treated with vehicle (V) or combined trametinib (1mg/kg body weight) and palbociclib (100 mg/kg body weight) (T/P) for 2 weeks (left). Right, flow cytometry analysis of NK cell numbers and degranulation in each condition (**a**) NK cell numbers PIP V, PIP TP and PIL TP, n=10; PIL V, n=9) and degranulation (n=5 independent mice per group). (**b**) NK cell numbers LIL V, n=3: LIL TP, n=5; LIP V, n=13; LIP TP, n=15 and degranulation LIP V, n=5; LIP TP, n=7 independent mice). Data represents pool of 3 independent experiments. **c**, *KPC1* PDAC or *KP1* LUAD cells expressing luciferase-GFP were injected orthotopically into the livers of 8-12 week old C57BL/6 female mice and treated as in (**a**) following tumor formation (left). Right, flow cytometry analysis of NK cell numbers Data represents pool of 2 independent experiments. (**c**) NK cell numbers and NK degranulation PILiver V, n= 9; PILiver TP, n=10 and LILiver V, n=8; LILiver TP, n=10 independent mice). **d**, Kaplan-Meier survival curve of C57BL/6 mice harboring *KPC1* PDAC tumors in pancreas (PIP) treated with vehicle or trametinib (1 mg/kg) and palbociclib (100 mg/kg) in the presence or absence of a NK1.1 depleting antibody (PK136; 250 ug) (V, n=5; TP and TP+αNK1.1, n=7 independent mice). **e**, Kaplan-Meier survival curve of C57BL/6 mice harboring *KPC1* PDAC tumors in lungs (PIL) and treated as in (**d**) (V, n=10; TP, n=9 and TP+αNK1.1, n=8 independent mice). **f**, Kaplan-Meier survival curve of C57BL/6 mice harboring *KP2* LUAD tumors in the lungs (LIL) and treated as in (**d**) (V, n=6; TP, n=7 and TP+αNK1.1, n=8 independent mice). **g**, Kaplan-Meier survival curve of C57BL/6 mice harboring *KP1* LUAD tumors in pancreas (LIP) and treated as in (**d**) (V, n=7; TP and TP+αNK1.1, n=8 independent mice). *P* values in **a-c** were calculated using two-tailed, unpaired Student’s t-test, and those in **d-g** calculated using log-rank test. Error bars, mean ± SEM.

Based on our previous findings we hypothesized that the pancreas TME may contribute to suppression of NK cell anti-tumor immunity following therapy-induced senescence. To test this, we took advantage of genetically similar KRAS mutant PDAC and LUAD cell lines that could be transplanted into different organs of syngeneic C57BL/6 mice to study the impact of the TME on senescence-driven immune responses. These included: (a) *KPC* PDAC tumor cell lines (*KPC1*, *KPC2*) derived from PDAC tumors in *Pdx1-Cre*; *Kras^LSL-G12D/wt^*;*Trp53^R172H/wt^* genetically engineered mouse models (GEMMs)^31^ and (b) *KP* LUAD cell lines (*KP1*, *KP2*) derived from lung tumors in *Kras^LSL-G12D/wt^;Trp53^flox/flox^* GEMMs administered an adenovirus expressing Cre-recombinase intratracheally^32^. *KPC* PDAC or *KP* LUAD cells were engineered to express luciferase and GFP (to track and isolate tumors *in vivo*) and then transplanted intravenously (i.v.) to form tumors in the lungs or injected directly into the pancreas of C57BL/6 mice (Fig. 1a,b). Additionally, PDAC and LUAD cells were also transplanted into the liver, a common site of metastasis for both tumor types (Fig. 1c). Following tumor formation, mice were treated with vehicle or T/P for two weeks to induce senescence (Fig. 1a–c). PDAC and LUAD tumors propagated in each organ had a similar tumor burden and disease histopathology, as well as anti-proliferative (Ki67) and on-target drug responses (pRb) to T/P treatment (Extended Data Fig. 1a,b). A comparable degree of senescence induction, as measured by senescence-associated β-galactosidase (SA-β-gal) and p21 expression in the tumor and RNA-sequencing (RNA-seq) analysis of a senescence gene set in FACS sorted GFP^+^ tumor cells, was also observed in tumors propagated in each organ following two-week T/P treatment (Extended Data Fig. 1b–d).

**Extended Data Fig. 2.**
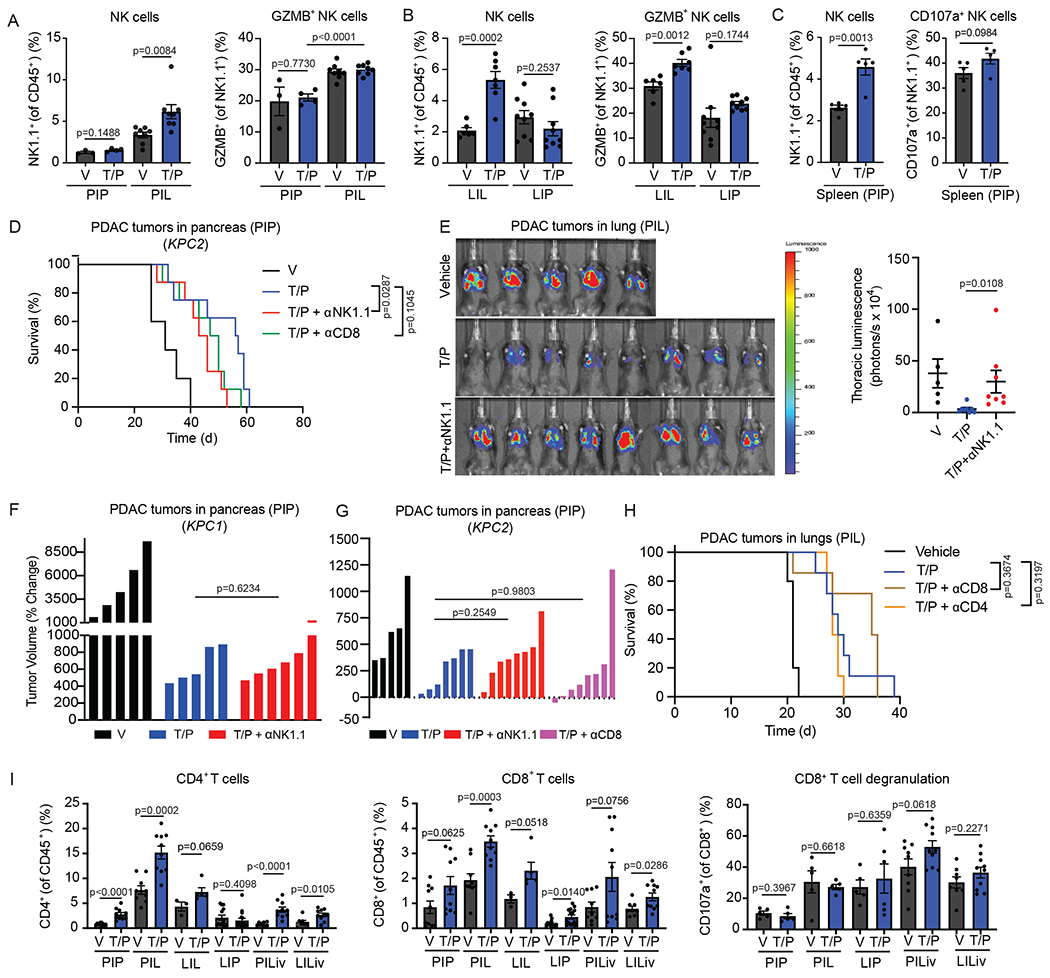
Suppression of NK immunity specific to pancreas TME following T/P-induced senescence. **a-b**, *KPC2* PDAC or *KP2* LUAD tumor cells expressing GFP were injected i.v. or orthotopically into the pancreas of 8-12 week old C57BL/6 female mice. Following tumor formation, mice were treated with vehicle (V) or combined trametinib (1mg/kg) and palbociclib (100 mg/kg) (T/P) for 2 weeks. Flow cytometry analysis of NK cell numbers and degranulation in PDAC (PIP, PIL) (**a**) and LUAD-derived tumors (LIL, LIP) (**b**) grown in different organs are shown (**a-b,** PIP V, n=3; PIP TP, n=4; PIL V and PIL TP, n=8 independent mice). **c**, Flow cytometry analysis of NK cell numbers and degranulation in spleens of mice with *KPC1*-derived PIP tumors treated as in (**a**) (n=5 independent mice per group). **d**, Kaplan-Meier survival curve of mice with *KPC2*-derived PIP tumors treated with vehicle, combined trametinib (1mg/kg) and palbociclib (100mg/kg), and/or depleting antibodies against NK1.1 (PK136; 250 μg) or CD8 (2.43; 200 μg) (**d,** V, n=5; TP; TP+αNK1.1 and TP+αCD8, n=8). **e,** IVIS images showing luciferase signaling in *KPC1*-derived PIL tumors following treatment as in (**a**). Right, quantification of total luminescence in the thoracic region (**e,** V, n=5; TP and TP+αNK1.1, n=8 independent mice). **f,** Waterfall plot of the response of *KPC1-*derived PIP tumors following 2 week treatment with vehicle, combined trametinib (1mg/kg) and palbociclib (100mg/kg), and/or an NK1.1 depleting antibody (PK136; 250 μg) (**f,** V and TP, n=5, TP+αNK1.1, n=6 independent mice). **g,** Waterfall plot of the response of *KPC2-*derived PIP tumors following 2 week treatment with vehicle, combined trametinib (1mg/kg) and palbociclib (100mg/kg), and/or an NK1.1 (PK136; 250 μg) or CD8 (2.43; 200 μg) depleting antibody (**g,** V, n=5; TP, n=7; TP+αNK1.1 and TP+αCD8 ,n=8 independent mice). **h**, Kaplan-Meier survival curve of mice with *KPC1*-derived PIL tumors treated with vehicle, combined trametinib (1mg/kg) and palbociclib (100mg/kg), and/or depleting antibodies against CD8 (2.43; 200 μg) or CD4 (GK1.5; 200 μg) (**h,** V, n=5; TP, TP+αNK1.1 and TP+αCD8, n=7 independent mice). **i**, Flow cytometry analysis of CD4^+^ and CD8^+^ T cell numbers and degranulation in *KPC1* PDAC (PIP, PIL, PILiver) and *KP1* LUAD-derived tumors (LIL, LIP, LILiver) grown in different organs and treated as in (**a**) (**i,** PIP V, PIP TP, PIL TP, n=10; PIL V, n=9; LIL V, n=3; LIL TP, n=5; LIP V, n=13; LIP TP, n=15; PILiver V, n=9; PILiver TP, n=10; LILiver V, n=8; LILiver TP, n=10 independent mice). Data represents pool of 3 independent experiments. *P* values in **a-c**, **e-g**, and **i** were calculated using two-tailed, unpaired Student’s t-test, and those in **d** and **h** were calculated using log-rank test. Error bars, mean ± SEM.

In line with our previous findings, T/P treatment led to increased NK cell accumulation and cytotoxicity, as marked by the degranulation markers CD107a and Granzyme B (GZMB), in LUAD tumors grown in the lungs (LIL) but not in PDAC tumors grown in the pancreas (PIP), despite peripheral NK cell expansion in adjacent spleens (Fig. 1a,b and Extended Data Fig. 2a–c)^19,20^. NK cell suppression was specific to the pancreas TME, as PDAC tumors grown in the lungs (PIL) or liver (PILiver) underwent NK cell surveillance following T/P-induced senescence (Fig. 1a,c and Extended Data Fig. 2a). Similarly, whereas LUAD tumors propagated in the liver (LILiver) were infiltrated with activated NK cells, those propagated in the pancreas (LIP) were not (Fig. 1b,c and Extended Data Fig. 2b). These tissue-specific changes in NK cell states were functionally relevant, as NK cell depletion with an NK1.1-targeting antibody (PK136) reduced the survival benefit of T/P treatment in mice bearing tumors in the lungs (LIL, PIL) but not those with tumors in the pancreas (LIP, PIP) (Fig. 1d–g), with the exception of *KPC2*-derived PIP tumors where there was a modest effect (Extended Data Fig. 2d). In agreement, NK cell depletion led to increased pancreatic tumor growth in the lung (PIL) but did not impact *KPC1* or *KPC2*-derived pancreatic tumor growth in the pancreas (PIP) following T/P treatment (Extended Data Fig. 2e–g). In contrast, T/P-induced senescence led to increased CD4^+^ and CD8^+^ T cells in nearly all tumors regardless of the resident organ, though the infiltrating T cells were not activated and T cell depletion studies indicated they did not contribute to anti-tumor immunity in the lung or pancreas TME (Extended Data Fig. 2d,h,i). Therefore, the pancreas TME leads to specific resistance to NK cell immune surveillance following therapy-induced senescence.

### The SASP is transcriptionally repressed in the pancreas TME

**Extended Data Fig. 3.**
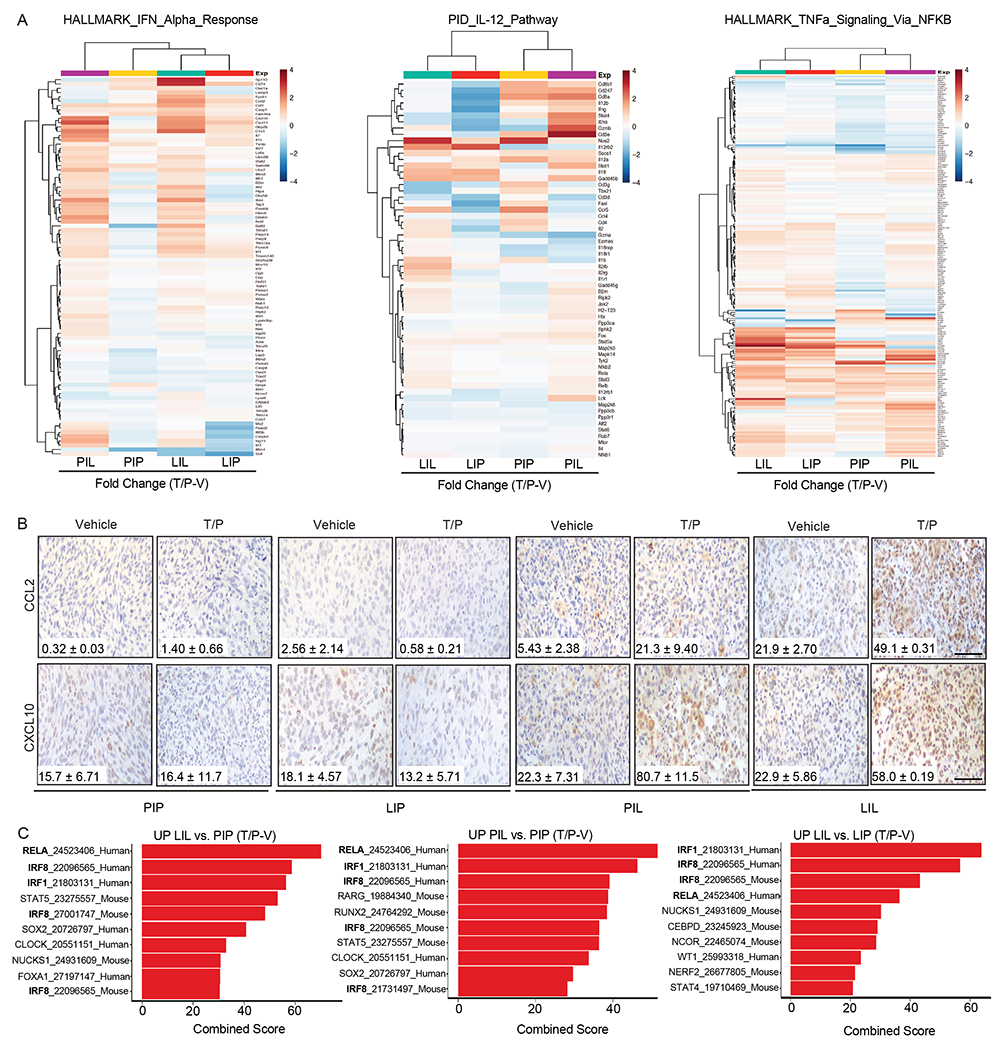
Repression of pro-inflammatory SASP gene expression specific to the pancreas TME following T/P treatment. **a**, Heatmaps showing fold change in IFNα (left), IL-12 (middle), and TNFα pathway genes (right) following T/P treatment in indicated tumor settings from RNA-seq data in Figure 2a (n=2-4 per group). **b**, IHC staining of indicated *KPC1* PDAC (PIP, PIL) and *KP1* LUAD (LIL, LIP) derived-tumors grown in different organs and treated with vehicle (V) or combined trametinib (1mg/kg) and palbociclib (100 mg/kg) (T/P) for 2 weeks. H-score quantification of CCL2 and CXCL10 staining intensity is shown inset (n=2-3 per group). Scale bars, 50μm. Error bars, mean + SEM. **c**, Transcription factor enrichment analysis showing transcriptional regulators whose targets are differentially expressed in tumors in the lungs (LIL, PIL) following T/P treatment.

**Fig. 2.**
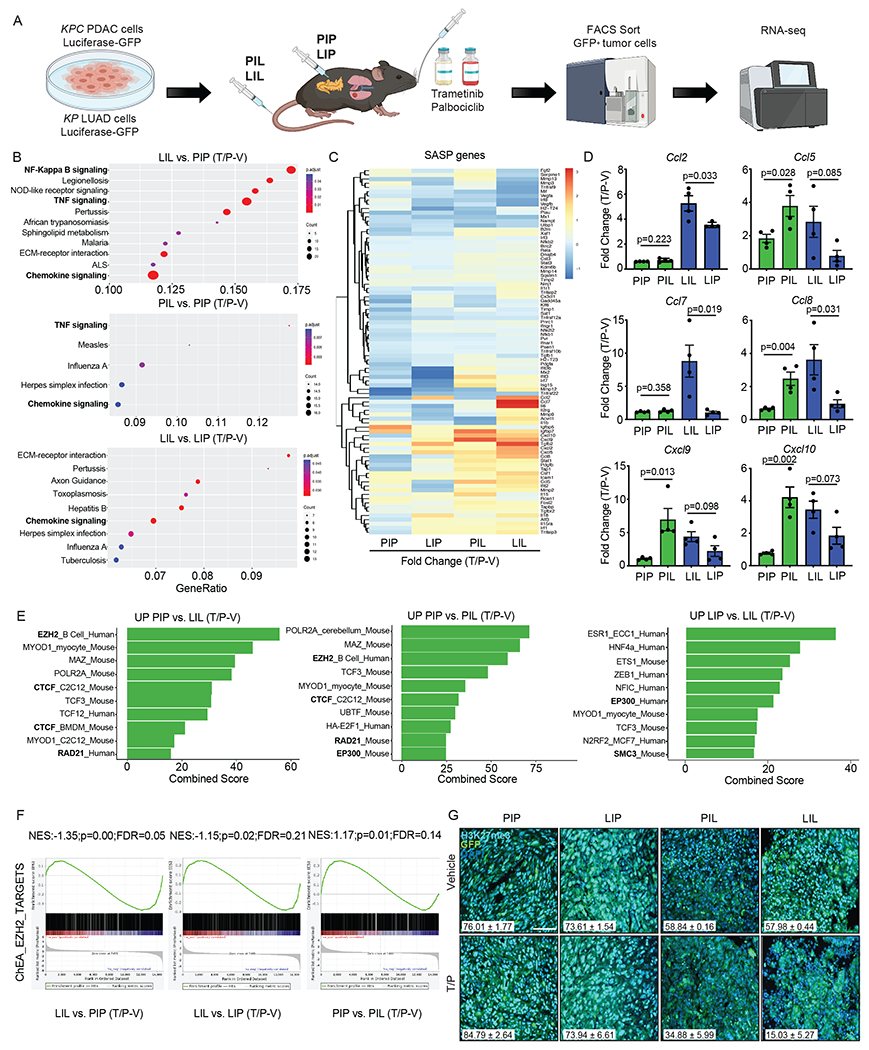
The pro-inflammatory SASP is transcriptionally and epigenetically repressed in the pancreas TME. **a**, *KPC1* PDAC or *KP1* LUAD tumor cells expressing luciferase-GFP were injected i.v. or orthotopically into the pancreas of 8-12 week old C57BL/6 female mice. Following tumor formation in the lungs or pancreas, mice were treated with vehicle (V) or combined trametinib (1mg/kg) and palbociclib (100 mg/kg) (T/P) for 2 weeks. GFP^+^ tumor cells were FACS sorted and extracted RNA subjected to RNA-seq analysis (n=2-4 per group). **b**, KEGG pathway analysis of pathways enriched in tumors in the lungs (LIL, PIL) compared to tumors in the pancreas (PIP, LIP) following T/P treatment. **c**, Heatmap showing fold change in SASP gene expression following T/P treatment in indicated tumor settings. **d**, Fold change in expression of select SASP chemokines following T/P treatment in indicated tumor settings (n=2-4 per group). Error bars, mean ± SEM. **e,** Transcription factor enrichment analysis showing transcriptional regulators whose targets are differentially expressed in tumors in the pancreas (PIP, LIP) following T/P treatment. **f,** Gene Set Enrichment Analysis (GSEA) of EZH2 transcriptional targets. NES, normalized enrichment score. **g,** Immunofluorescence staining of indicated *KPC1* PDAC (PIP, PIL) and *KP1* LUAD (LIL, LIP) derived-tumors grown in different organs and treated with vehicle (V) or combined trametinib (1mg/kg) and palbociclib (100 mg/kg) (T/P) for 2 weeks. Quantification of the percentage GFP^+^ (green) tumor cells expressing H3K27me3 (cyan) is shown inset (PIP V; PIP TP; PIL TP; LIL TP and LIP TP, n=3, PIL V; LIL V and LIP V, n=2 independent tumors). Scale bar, 50μm. *P* values in **b** were calculated using two-sided, hypergeometric test and those in **f** using two-sided, Kolmogorov-Smirnov test.

We next performed RNA-seq on GFP-labeled PDAC and LUAD cells FACS sorted from lung or pancreas tumors following T/P treatment to determine the impact of the TME on signaling in senescent tumor cells (Fig. 2a and Supplementary Table 1). T/P treatment led to significant enrichment of inflammatory pathways related to NF-κB, TNF, and chemokine signaling, as well as type I interferon and IL-12 pathways known to activate innate and in particular NK cell immunity, in PDAC and LUAD cells in the lungs (PIL, LIL) as compared to those in the pancreas (LIP, PIP) (Fig. 2b, Extended Data Fig. 3a, and Supplementary Tables 2–17). A subset of pro-inflammatory SASP genes were significantly upregulated following T/P-induced senescence in PDAC and LUAD cells in the lung TME (Fig. 2c and Supplementary Tables 18–21). These included a number of SASP-related chemokines known to regulate the chemotaxis of NK cells and T cells into tumors, including CCL2, CCL5, CCL7, CCL8, CXCL9, and CXCL10, that were induced at both the gene expression and protein level following T/P treatment of tumors in the lung but not pancreas TME (Fig. 2d and Extended Data Fig. 3b)^33^.

Senescence induction is associated with dynamic transcriptional and chromatin changes. Rb and p53-regulated pathways mediate repression of cell cycle genes^34,35^. In addition, a number of other transcription factors and regulators, including NF-κB, C/EBPβ, cGAS-STING, JAK/STATs, and NOTCH, lead to activation of SASP programs^36–39^. These transcriptional changes are accompanied by dramatic remodeling of the chromatin landscape, with Rb enabling repressive H3K9me3-mediated chromatin compaction at cell cycle genes^35^, and BRD4 facilitating H3K27Ac-mediated enhancer activation at SASP loci^40^. Transcription factor enrichment analysis demonstrated that transcriptional targets of NF-κB and its p65 subunit RELA, which we have shown to be important activators of the SASP following T/P-induced senescence^19,20^, as well as targets of interferon regulatory factors (IRFs) that drive interferon production downstream of STING pathway activation, were induced preferentially in tumors propagated in the lung TME (Extended Data Fig. 3c and Supplementary Table 22). Interestingly, regulators of 3D chromatin topology and DNA looping (CTCF, RAD21, SMC3), as well as histone modifications and chromatin compaction (EZH2, p300), were enriched in tumors within the pancreas TME (Fig. 2e and Supplementary Table 22). These findings suggested the possibility that chromatin remodeling within tumors in the pancreas TME may lead to transcriptional repression of the SASP.

**Extended Data Fig. 4.**
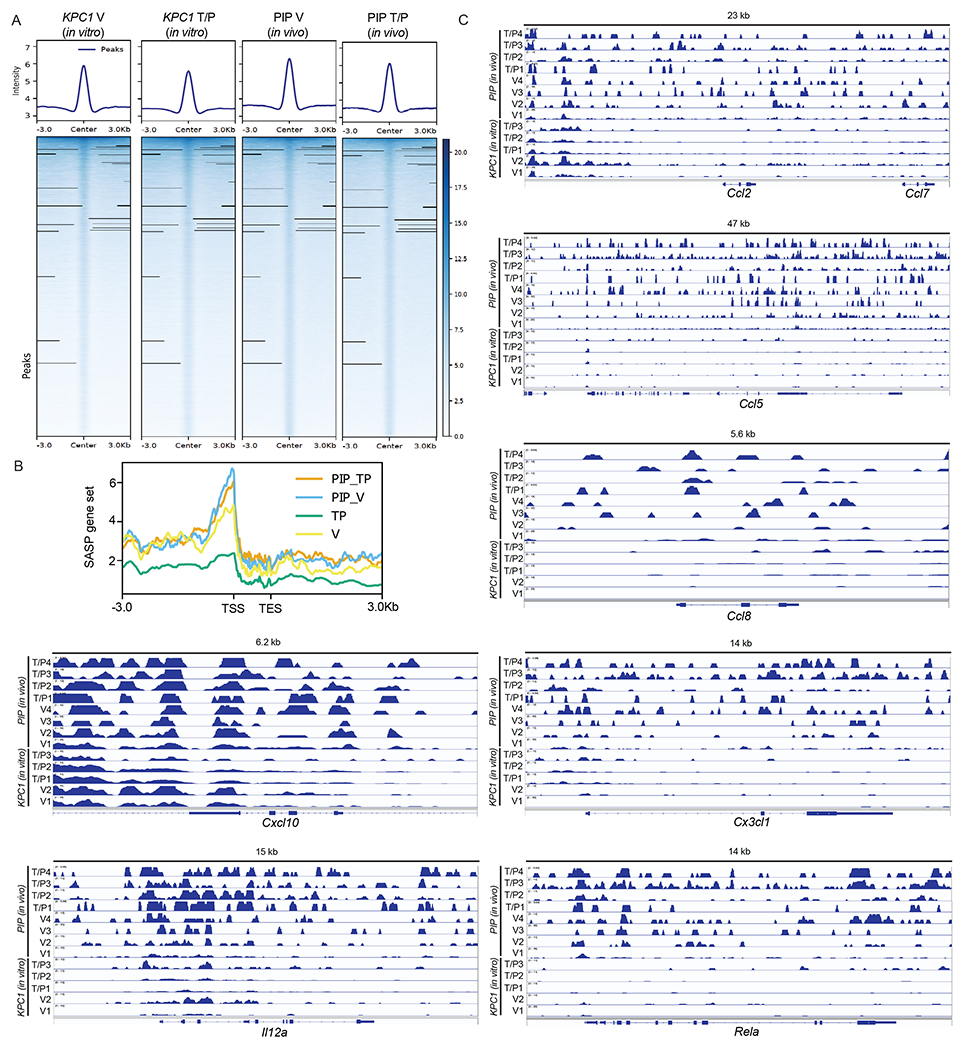
Tumors in the pancreas TME are enriched for H3K27me3 repressive chromatin marks at SASP gene loci. **a**, Heatmaps of normalized genome-wide H3K27me3 signaling intensities of consensus peaks from CUT&Tag analysis of *KPC1* cells treated with vehicle or trametinib (25 nM) and palbociclib (500 nM) *in vitro* for 8 days, or *KPC1* cells FACS sorted from transplanted PDAC tumors in C57BL/6 mice treated with vehicle or trametinib (1 mg/kg) and palbociclib (100 mg/kg) for 2 weeks (n=2-4 per group). **b,** Normalized H3K27me3 peak intensities of 87 SASP genes (see Table 47) from CUT&Tag analysis samples in (**a**) (n=2-4 per group). **c**, Genome browser tracks showing H3K27me3 occupancy at pro-inflammatory SASP gene loci from CUT&Tag analysis samples in (**a**) (n=2-4 per group).

**Extended Data Fig. 5.**
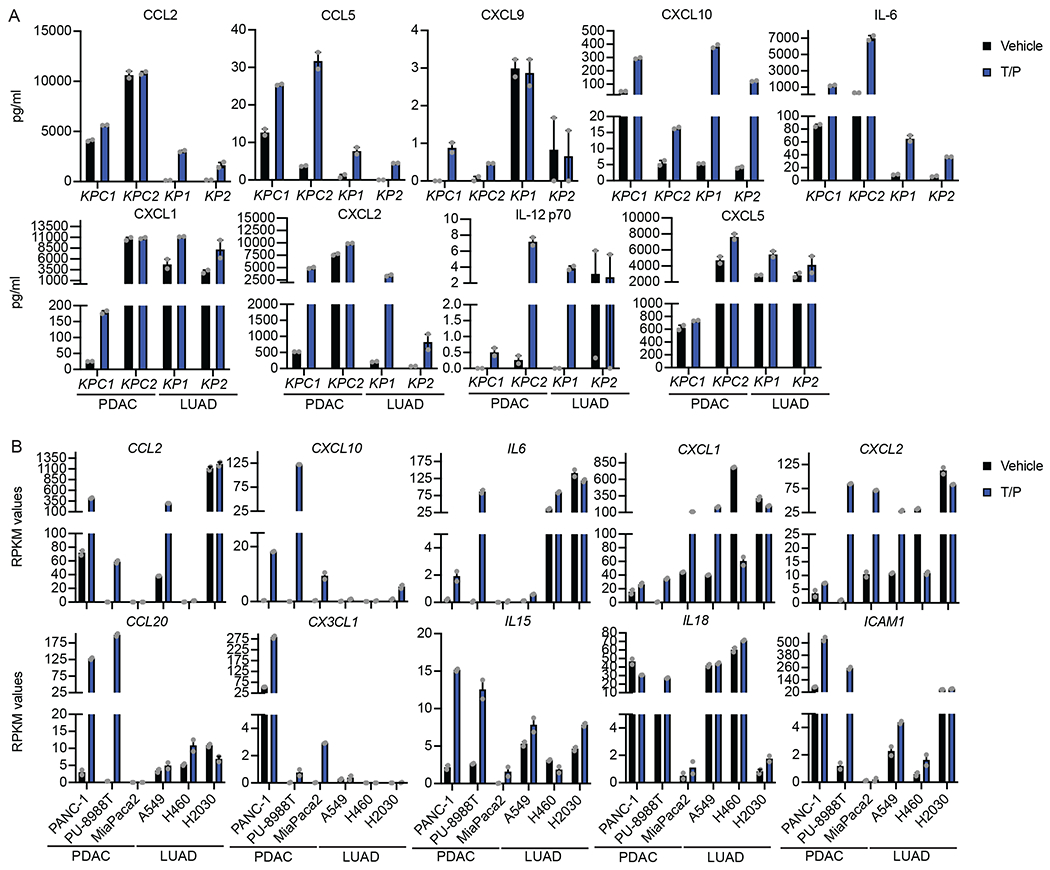
PDAC and LUAD tumor cells have a similar pro-inflammatory SASP response to T/P-induced senescence *in vitro*. **a**, Cytokine array analysis of pro-inflammatory SASP genes in murine PDAC and LUAD cell lines treated with vehicle or combined trametinib (25 nM) and palbociclib (500nM) for 8 days (n=2 per group). #, outside the detectable limit. **b**, Normalized expression levels of pro-inflammatory SASP genes in human PDAC and LUAD cell lines following treatment as in (**a**) from analysis of RNA-seq data generated in Ruscetti et al. (2018)^19^ (n=2 per group).

EZH2 is a member of the polycomb repressor complex 2 (PRC2) with methyltransferase activity that mediates transcriptional gene repression through H3K27 trimethylation (H3K27me3)^41^. EZH2 has previously been shown to regulate the expression of *CDKN2A* (i.e. p16) and other senescence and SASP-related genes^42–44^. Indeed, we found expression of EZH2 target genes significantly enriched, and H3K27me3 levels dramatically increased, in tumors propagated in the pancreas as compared to those in the lungs (Fig. 2f,g). CUT&Tag analysis^45^ revealed that H3K27me3 peaks were enriched globally and at SASP-related gene loci in *KPC1* PDAC tumor cells FACS sorted from tumors grown in the pancreas (PIP) *in vivo* as compared to *KPC1* PDAC cells grown *in vitro*, including for instance at *Ccl2, Ccl5, Ccl7, Ccl8,* and *Cxcl10* loci that are transcriptionally repressed in the PDAC TME (Extended Data Fig. 4 and Supplementary Tables 23–26). Moreover, whereas T/P treatment reduced H3K27me3 marks at pro-inflammatory SASP genes in *KPC1* PDAC cells and induced their expression to a similar degree in KRAS mutant mouse and human pancreatic and lung cancer cell lines *in vitro*, T/P-induced senescence did not turn on SASP gene expression transcriptionally or reduce repressive H3K27me3 marks in these tumors cells when grown in the pancreas TME *in vivo* (Extended Data Fig. 4 and 5 and Supplementary Tables 23–26). Thus, pro-inflammatory SASP genes known to modulate immune cell function and regulate NK cell immune surveillance are both transcriptionally and epigenetically repressed in the pancreas TME following therapy-induced senescence.

### SMA^+^ fibroblasts constrain SASP-mediated NK and T cell immunity

**Fig. 3.**
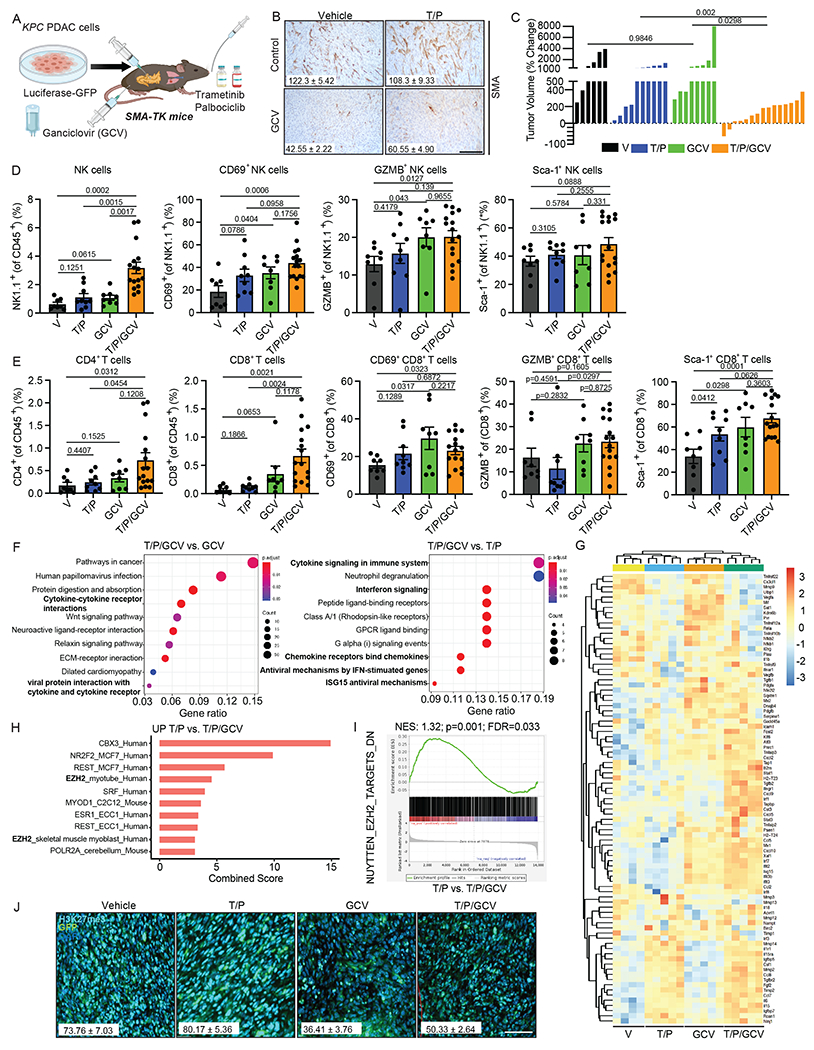
SMA^+^ fibroblasts in the pancreas TME constrain SASP-mediated NK and T cell immunity and promote EZH2 activation in PDAC. **a**, Schematic of *KPC* PDAC syngeneic orthotopic transplantation into 8-16 week old male and female *SMA**-TK* mice and treatment regimens. **b**, Immunohistochemical (IHC) staining of *KPC1* orthotopic PDAC tumors propagated in *SMA-TK* mice treated with vehicle, trametinib (1 mg/kg) and palbociclib (100 mg/kg), and/or ganciclovir (GCV) (50 mg/kg) for 2 weeks. Quantification of number of SMA^+^ cells per field is shown inset (**b**, V, n=5; TP, n=4; GCV, n=4; and TP/GCV, n=5 independent tumors). Scale bar, 50μm. **c**, Waterfall plot of the response of *KPC1* orthotopic PDAC tumors propagated in *SMA-TK* mice to treatment as in (**b**) (**c**, V, n=6; TP, n=10; GCV, n=8, and TP/GCV, n=15 independent mice). Data represents pool of 2 independent experiments. **d-e**, Flow cytometry analysis of NK (**d**) and T cell (**e**) numbers and activation markers in *KPC1* orthotopic PDAC tumors propagated in *SMA-TK* mice following treatment as in (**b**) (n=8-16 per group). (**d-e**, V, n=8; TP, n=9; GCV, n=8, and TP/GCV, n=16 independent mice). Data represents pool of 2 independent experiments. Error bars, mean ± SEM. **f**, KEGG (left) and REACTOME (right) pathway analysis of RNA-seq data generated from FACS sorted GFP^+^ tumor cells from SMA-TK mice harboring *KPC1* orthotopic PDAC tumors and treated as in (**b**) (n=4-5 per group). **g**, Heatmap of RNA-seq analysis of SASP gene expression in PDAC cells from *KPC1* orthotopic PDAC tumors propagated in *SMA-TK* mice and treated as in (**b**) (n=4-5 per group). **h**, Transcription factor enrichment analysis showing transcriptional regulators whose targets are differentially expressed in tumor cells from *KPC1* orthotopic PDAC propagated in *SMA-TK* mice treated with T/P alone compared with combined T/P/GCV treatment. **i**, Gene Set Enrichment Analysis (GSEA) of EZH2 transcriptional targets. NES, normalized enrichment score. **j**, Immunofluorescence staining of *KPC1* orthotopic PDAC tumors propagated in *SMA-TK* mice treated as in (**b**). Quantification of the percentage GFP^+^ (green) tumor cells expressing H3K27me3 (cyan) is shown inset (n=2 per group). Scale bar, 50μm. *P* values in **c**-**e** were calculated using two-tailed, unpaired Student’s t-test, **f** using two-sided, hypergeometric test, and **i** using two-sided, Kolmogorov-Smirnov test.

A hallmark of PDAC that distinguishes it from other solid tumor types is its fibrotic and desmoplastic stroma that arises, in part, through secretion of extracellular matrix (ECM) proteins by SMA^+^ myofibroblasts^46^. To assess whether prevalent SMA^+^ fibroblasts are responsible for SASP inhibition and subsequent NK and T cell suppression specific to the PDAC TME, we took advantage of a previously characterized *SMA-TK* mouse model^47^ where SMA^+^ fibroblasts can be selectively depleted upon administration of ganciclovir (GCV). Following transplantation of *KPC1* cells into the pancreas and PDAC formation in *SMA-TK* mice, animals were treated with vehicle, T/P, and/or GCV for two weeks to determine the impact SMA depletion on immune responses and SASP output (Fig. 3a,b).

T/P-induced senescence in combination with GCV-mediated SMA^+^ fibroblast depletion led to significantly decreased tumor growth and increased infiltration of NK and CD4^+^ and CD8^+^ T cells and their expression of activation (CD69, Sca-1) and cytotoxicity (GZMB) markers compared to T/P treatment alone (Fig. 3c–e). Pathway analysis of RNA-seq data from FACS sorted tumor cells revealed enriched expression of genes related to cytokine, chemokine, and interferon signaling in PDAC tumors treated with combined T/P and GCV compared with those treated with T/P or GCV alone (Fig. 3f and Supplementary Tables 27–32). GCV-mediated SMA^+^ fibroblast depletion also led to increased expression of pro-inflammatory SASP genes following T/P-induced senescence, including key SASP factors such as *Ccl2/5/8*, *Cxcl9/10*, and *Il15* that we have shown to be repressed in the PDAC TME (Fig. 3g and Supplementary Table 33). Deeper analysis revealed that EZH2 was among the top transcriptional regulators whose targets were enriched in T/P compared to T/P/GCV treated PDAC tumors, with fibroblast depletion leading to reduced expression of EZH2 target genes and H3K27me3 levels (Fig. 3h–j and Supplementary Table 34). Collectively, these results demonstrate that SMA^+^ fibroblasts contribute to SASP and NK and T cell suppression in the pancreas TME, and suggest that this may be mediated in part through EZH2-driven transcriptional repression of inflammatory signaling in tumor cells.

### EZH2 blockade reactivates the pro-inflammatory SASP in PDAC

**Extended Data Fig. 6.**
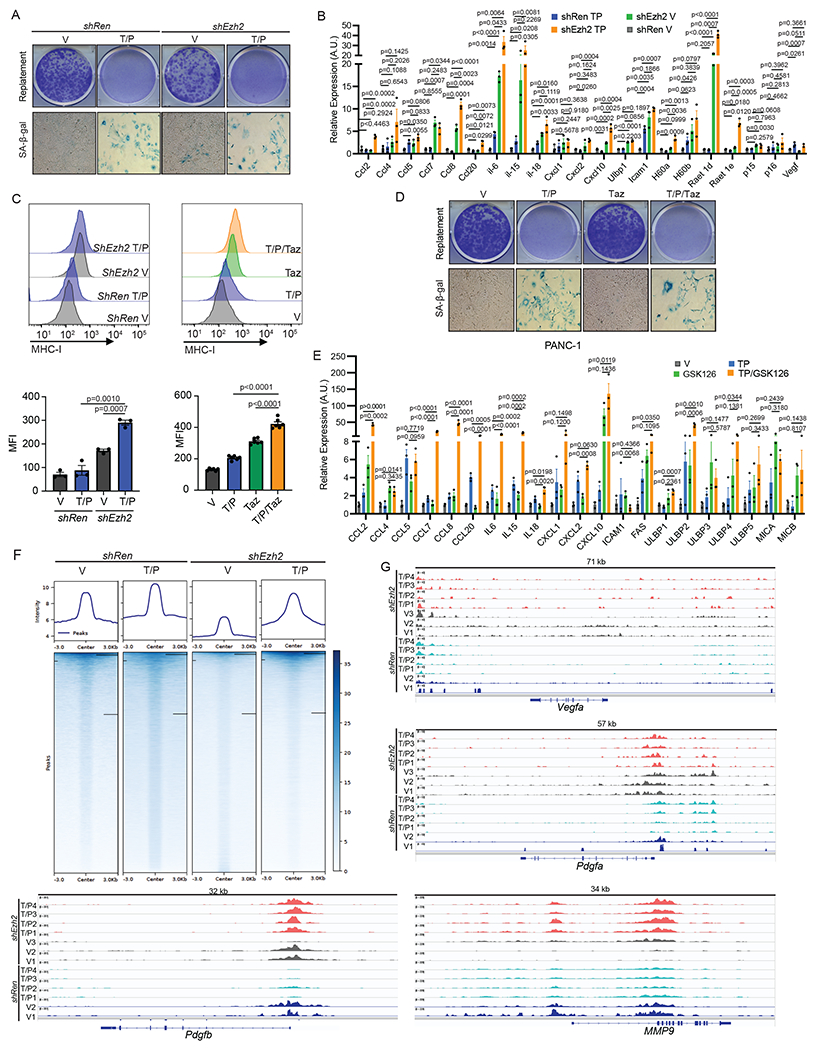
Suppression of EZH2-mediated H3K27me3 induces pro-inflammatory SASP and immunomodulatory cell surface molecules following T/P treatment in PDAC cells. **a**, Representative clonogenic assay images (from 3 biological replicates) of *KPC1* PDAC cells harboring *shRen* or *shEzh2* shRNAs replated in the absence of drugs after an 8-day pre-treatment with vehicle or combined trametinib (25 nM) and palbociclib (500 nM) (top). Bottom, representative SA-β-gal staining (from 3 biological replicates) of *KPC1* PDAC cells harboring *shRen* or *shEzh2* shRNAs and treated with vehicle or combined trametinib (25 nM) and palbociclib (500 nM) for 8 days. **b**, qRT-PCR analysis of senescence and SASP gene expression in *KPC1* PDAC cells harboring *shRen* or *shEzh2* shRNAs treated with vehicle or combined trametinib (25 nM) and palbociclib (500 nM) for 8 days (n=3 biological replicates per group). A.U., arbitrary units. **c**, Representative histograms (top) and quantification of mean fluorescent intensity (MFI) of MHC-I (H-2k^b^) expression (bottom) on *KPC1* PDAC cells harboring *shRen* or *shEzh2* shRNAs (left) or parental *KPC1* PDAC cells (right) treated with vehicle, combined trametinib (25 nM) and palbociclib (500nM), and/or tazemetostat (5 μM) for 8 days (shRen V, shRen TP, shEzh2 V, shEzh2 TP, n=3; V, TP, TAZ, TP/TAZ, n=6 independent mice). **d**, Representative clonogenic assay images (from 3 biological replicates) of *KPC1* PDAC cells replated in the absence of drugs after an 8-day pre-treatment with vehicle, combined trametinib (25 nM) and palbociclib (500 nM), and/or tazemetostat (5 μM) (top). Bottom, representative SA-β-gal staining (from 3 biological replicates) of *KPC1* PDAC cells treated with vehicle, combined trametinib (25 nM) and palbociclib (500 nM), and/or tazemetostat (5 μM) for 8 days. **e**, qRT-PCR analysis of SASP gene expression in human PANC-1 PDAC cells treated with vehicle, trametinib (25 nM), palbociclib (500 nM), and/or GSK126 (1 μM) for 8 days (n=3 per group). A.U., arbitrary units. **f**, Heatmaps of normalized genome-wide H3K27me3 signaling intensities from CUT&Tag analysis of *KPC1* PDAC cells harboring *Ren* or *Ezh2* shRNAs treated with vehicle or trametinib (25 nM) and palbociclib (500 nM) for 8 days (n=2-4 per group). **g**, Genome browser tracks showing H3K27me3 occupancy at pro-angiogenic SASP gene loci (n=2-4 per group). *P* values in **b, c, and e** were calculated using two-tailed, unpaired Student’s t-test. Error bars, mean ± SEM.

**Fig. 4.**
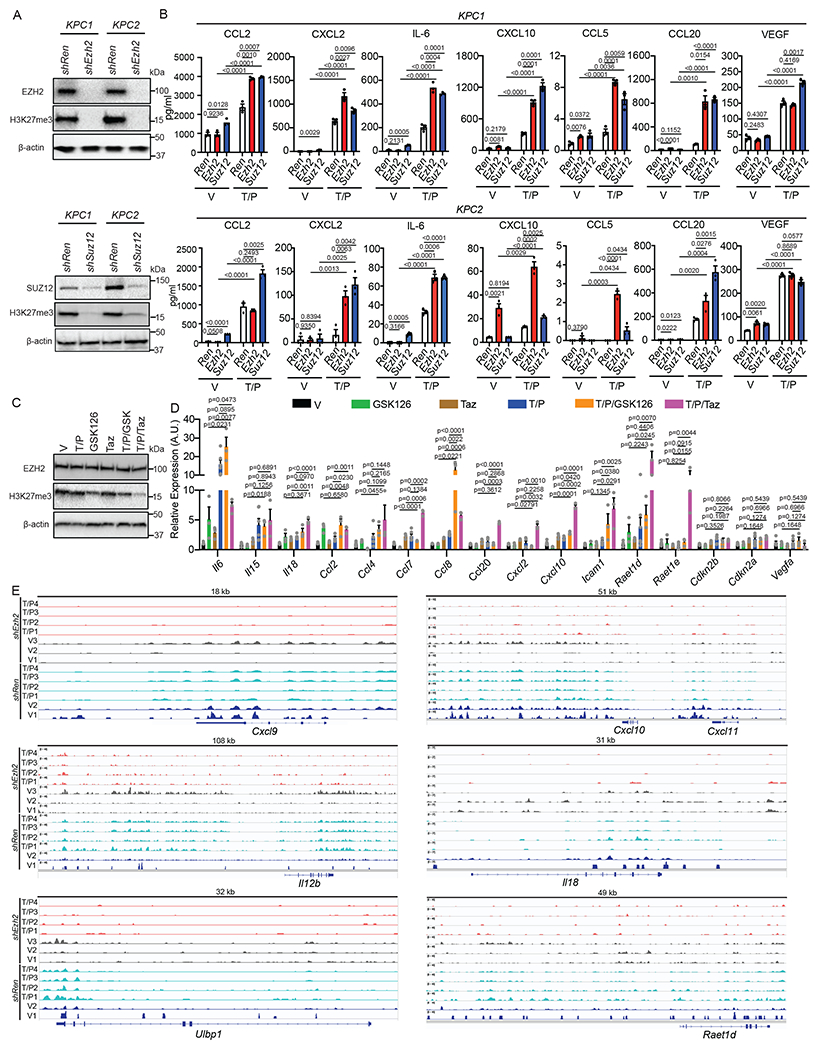
Targeting EZH2 expression or its methyltransferase activity reactivates the pro-inflammatory SASP in PDAC. **a**, Immunoblots of *KPC1* and *KPC2* PDAC cells harboring *Renilla* (*Ren*), *Ezh2*, or *Suz12* shRNAs. **b**, Cytokine array results from *KPC1* and *KPC2* PDAC cells with indicated shRNAs treated for 8 days with vehicle or trametinib (25 nM) and palbociclib (500 nM) (n=3 per group). **c**, Immunoblots of *KPC1* PDAC cells treated with vehicle, trametinib (25 nM), palbociclib (500 nM), GSK126 (1 μM), and/or tazemetostat (5 μM) for 8 days. **d**, qRT-PCR analysis of senescence and SASP gene expression in *KPC1* PDAC cells treated as in **c** for 8 days (**d,** V, n=6; TP, n=6 for all genes except Cxcl10 where n=3, GSK126; TP/GSK126; TAZ and TP/TAZ, n=3 biological replicates). A.U., arbitrary units. Data represents pool of 2 independent experiments. **e**, Genome browser tracks from CUT&Tag analysis showing H3K27me3 occupancy at pro-inflammatory SASP gene loci in *KPC1* PDAC cells harboring *Ren* or *Ezh2* shRNAs treated with vehicle or trametinib (25 nM) and palbociclib (500 nM) for 8 days (n=2-4 per group). *P* values in **b** and **d** were calculated using two-tailed, unpaired Student’s t-test. Error bars, mean ± SEM.

Based on our findings that EZH2 target genes were differentially expressed and repressive H3K27me3 marks were enriched at pro-inflammatory SASP gene loci in the PDAC TME (Fig. 2e–g, 3h–j, and Extended Data Fig. 4), we hypothesized that targeting EZH2 or its methyltransferase activity could overcome epigenetic silencing of the SASP and subsequent suppression of cytotoxic lymphocyte immunity in PDAC. Short hairpin RNAs (shRNAs) were generated that could potently suppress EZH2 or SUZ12, another PRC2 complex component that interacts with EZH2, and subsequent H3K27me3 levels in our *KPC* PDAC cell lines (Fig. 4a). EZH2 knockdown had no impact on senescence-induced growth arrest or expression of SA-β-gal or other senescence-related genes (e.g. *Cdkn2a*, *Cdkn2b*) following T/P treatment in *KPC* PDAC cells (Extended Data Fig. 6a,b). In contrast, SASP-related pro-inflammatory cytokines (e.g. IL-6, IL-15, IL-18) and chemokines (CCL2/5/7/8/20, CXCL2/10) important for cytotoxic lymphocyte immunity were upregulated at the gene expression and protein secretion levels in *KPC* PDAC cells harboring *Ezh2* or *Suz12*-targeting shRNAs as compared to those harboring a control *Renilla* (*Ren*) shRNA alone and to a significantly greater extent in combination with T/P treatment (Fig. 4b and Extended Data Fig. 6b). Immunomodulatory cell surface proteins associated with the SASP, including cell adhesion molecules (ICAM-1) important for tumor-lymphocyte synapses, stress ligands that bind and stimulate the activating NKG2D receptor on NK cells (ULBP1, H60a, RAET1D/E), and MHC-I necessary for antigen presentation to T cells, were also strongly upregulated following therapy-induced senescence and EZH2 knockdown (Extended Data Fig. 6b,c).

This increased expression of inflammatory molecules observed following EZH2 inhibition was dependent on its methyltransferase activity, as treatment with the well-characterized EZH2 methyltransferase inhibitors GSK126 and tazemetostat (Taz) reduced H3K27me3 levels and led to induction pro-inflammatory SASP factors and immunomodulatory cell surface proteins without impacting senescence-associated cell cycle arrest following T/P treatment in both murine KPC as well as human PANC-1 PDAC cell lines (Fig. 4c,d and Extended Data Fig. 6c–e). In addition, we performed CUT&Tag analysis on *shRen* and *shEzh2 KPC1* cells to determine whether loss of repressive H3K27me3 marks at SASP gene loci was responsible for SASP transcriptional reprogramming following EZH2 targeting. EZH2 blockade led to a global reduction in genome-wide H3K27me3 levels, and pro-inflammatory SASP and NK cell ligand gene loci harboring H3K27me3 peaks at baseline, including *Cxcl9*, *Cxcl10, Cxcl11, Il12*, *Il18*, *Ulbp1*, and *Raet1d*, demonstrated synergistic H3K27me3 loss with combined EZH2 knockdown and T/P-induced senescence (Fig. 4e, Extended Data Fig. 6f, and Supplementary Tables 35–39). In contrast, H3K27me3 marks at pro-angiogenic SASP genes such as *Vegfa, Pdgfa, Pdgfb,* and *Mmp9* that were reduced following T/P treatment in the control *shRen* setting remained unchanged or even increased at these loci following T/P treatment in the *shEzh2* setting (Extended Data Fig. 6g and Supplementary Tables 35–39), suggesting EZH2 suppression preferentially impacted H3K27 trimethylation at a select group of pro-inflammatory SASP genes. Therefore, suppression of EZH2 methyltransferase activity in combination with therapy-induced senescence can synergistically reverse the epigenetic repression and promote the transcriptional activation of specific pro-inflammatory SASP genes in PDAC.

### EZH2 knockdown leads to NK and T cell-mediated PDAC control

**Extended Data Fig. 7.**
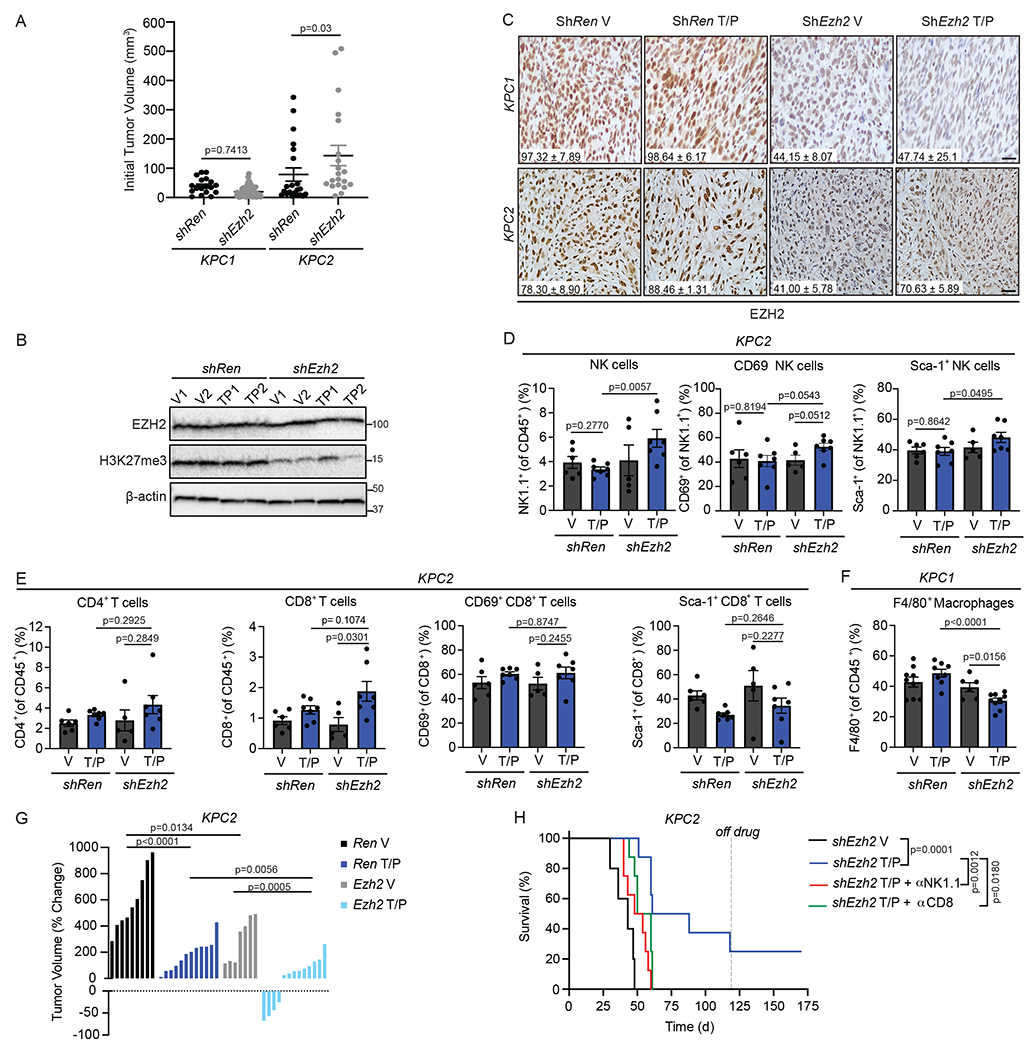
EZH2 knockdown in the *KPC2* PDAC orthotopic transplant model potentiates anti-tumor NK and CD8^+^ T cell immunity and long-term tumor regressions following T/P treatment. **a**, Ultrasound quantification of initial PDAC tumor volume 1-week post-transplantation of *KPC1* or *KPC2* cells harboring *shRen* or *shEzh2* shRNAs into 8-12 week old C57BL/6 female mice prior to enrollment in treatment cohorts (n=18-75 per group). Data represents pool of 6 independent experiments. **b,** Immunoblots of *shRen* or *shEzh2 KPC1* orthotopic PDAC tumors treated with vehicle or trametinib (1 mg/kg) and palbociclib (100 mg/kg) for 2 weeks. **c,** IHC staining of *KPC1* and *KPC2* orthotopic PDAC tumors harboring *shRen* or *shEzh2* shRNAs treated as in (**b**). H-score quantification of EZH2 expression is shown inset (n=2-3 per group). Scale bars, 50μm. **d-e**, Flow cytometry analysis of NK cell (**d**) and T cell (**e**) numbers and activation markers in *KPC2* orthotopic PDAC tumors harboring indicated shRNAs treated as in (**b**) (**d,e** shRen V, n=6; shRen TP, n=7; shEzh2 V, n=5; shEzh2 TP, n=7 independent mice). **f**, Flow cytometry analysis of F4/80^+^ macrophages in *KPC1* orthotopic PDAC tumors harboring indicated shRNAs treated as in (**b**) (**f,** shRen V, n=9; shRen TP, n=8; shEzh2 V, n=6; shEzh2 TP, n=9 independent mice). Data represents pool of 2 independent experiments. **g**, Waterfall plot of the response of *KPC2* orthotopic PDAC tumors harboring indicated shRNAs to treatment as in (**b**) (**g,** shRen V, n=9; shRen TP, n=12; shEzh2 V, n=7; shEzh2 TP, n=13 independent mice). Data represents pool of 2 independent experiments. **h**, Kaplan-Meier survival curve of mice with *shEzh2 KPC2* orthotopic PDAC tumors treated with vehicle, combined trametinib (1mg/kg) and palbociclib (100mg/kg), and/or depleting antibodies against NK1.1 (PK136; 250 μg) or CD8 (2.43; 200 μg) (**h,** shEzh2 V, n=5; shEzh2 TP, n=8; shEzh2 TP+αNK1.1, n=8; shEzh2 TP+αCD8, n=8 independent mice). Dotted line indicates timepoint when mice were taken off of treatment. *P* values in **a** were calculated using One-way ANOVA followed by Tukey’s multiple comparison test, **d-g** using two-tailed, unpaired Student’s t-test, and **h** using log-rank test. Error bars, mean ± SEM.

**Fig. 5.**
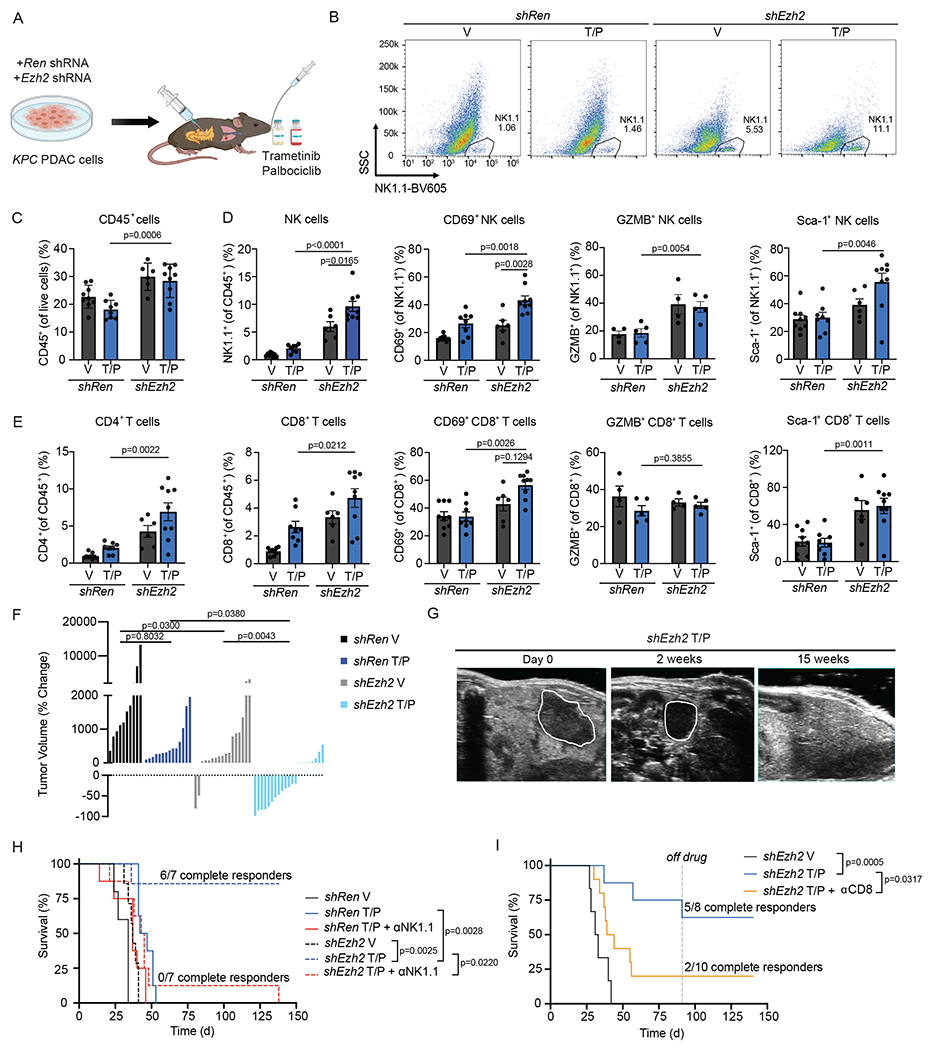
EZH2 blockade activates NK and T cell-mediated long-term tumor control following therapy-induced senescence in PDAC models. **a**, Schematic of *KPC* PDAC syngeneic orthotopic transplant model in 8-12 week old C57BL/6 female mice and treatment regimens. **b**, Representative flow cytometry plots of CD45^+^CD3^−^NK1.1^+^ NK cells in *KPC1* orthotopic PDAC tumors harboring indicated shRNAs from mice treated with vehicle (V) or combined trametinib (1mg/kg) and palbociclib (100mg/kg) (T/P) for 2 weeks. SSC, side scatter. **c-e**, Flow cytometry analysis of total CD45^+^ immune cells (**c**), NK cell numbers and activation markers (**d**), and T cell numbers and activation markers (**e**) in *KPC1* orthotopic PDAC tumors harboring indicated shRNAs following treatment as in (**b**) (**c-e**, shRen V, n=9; shRen TP, n=8; shEzh2 V, n =6; shEzh2 TP, n=9 independent mice). Data represents pool of 2 independent experiments. **f**, Waterfall plot of the response of *KPC1* orthotopic PDAC tumors with indicated shRNAs to treatment as in (**b**) (**f**, shRen V n=10, shRen TP, n=14; shEzh2 V, n =17; shEzh2 TP, n=21 independent mice) . Data represents pool of 3 independent experiments. **g**, Representative ultrasound images of a *shEzh2 KPC*1 orthotopic PDAC tumor prior to treatment and after 2 or 15 weeks of treatment with combined trametinib (1 mg/kg) and palbociclib (100 mg/kg). PDAC tumors are outlined in white. **h**, Kaplan-Meier survival curve of mice with *KPC*1 orthotopic PDAC tumors harboring indicated shRNAs treated with vehicle, combined trametinib (1mg/kg) and palbociclib (100mg/kg), and/or an NK1.1 depleting antibody (PK136; 250 μg) (**h**, shRen V, n=5; shRen TP; shRen TP+αNK1.1 and shEzh2 TP+αNK1.1, n=8; shEzh2 V and shEzh2 TP, n=7 independent mice) . **i,** Kaplan-Meier survival curve of mice with *shEzh2 KPC*1 orthotopic PDAC tumors treated with vehicle, combined trametinib (1mg/kg) and palbociclib (100mg/kg), and/or a CD8 depleting antibody (2.43; 200 μg) (**i,** shEzh2 V, n=6; shEzh2 TP, n=8 and shEzh2 TP+αCD8, n=10 independent mice). Dotted line indicates timepoint when mice were taken off of treatment. *P* values in **c-f** were calculated using two-tailed, unpaired Student’s t-test, and those in **h** and **i** calculated using log-rank test. Error bars, mean ± SEM.

To understand the impact of EZH2 suppression on senescence-mediated anti-tumor immunity in PDAC, we transplanted *KPC1* or *KPC2* PDAC cells harboring control Renilla (*shRen*) or EZH2-targeting (*shEzh2*) shRNAs orthotopically into the pancreas of C57BL/6 mice (Fig. 5a). Transplanted *shEzh2* PDAC cells formed tumors at a similar rate as *shRen* PDAC cells and maintained EZH2 and H3K27me3 knockdown *in vivo* (Extended Data Figs. 7a–c). Following tumor formation as confirmed by ultrasound imaging, mice were randomized into treatment groups where they received T/P or a vehicle control (Fig. 5a). Immunophenotyping by multi-parametric flow cytometry analysis following two-week treatment revealed significant changes in lymphocyte numbers and activity. T/P treatment in the setting of EZH2 knockdown led to increased total leukocyte infiltration, including enhanced NK cell accumulation and activity as marked by higher levels of early activation (CD69, Sca-1) and cytotoxicity (GZMB) markers on NK cells (Fig. 5b–c and Extended Data Fig. 7d). Increases in CD4^+^ and CD8^+^ T cell numbers and their expression of CD69 and Sca-1 were also observed in *KPC1* (but not *KPC2*)-derived shEzh2 PDAC lesions upon T/P treatment (Fig. 5e and Extended Data Fig. 7e). This expansion in activated lymphocytes following T/P-induced senescence and EZH2 blockade was also accompanied by a decrease in F4/80^+^ macrophages (Extended Data Fig. 7f).

**Extended Data Fig. 8.**
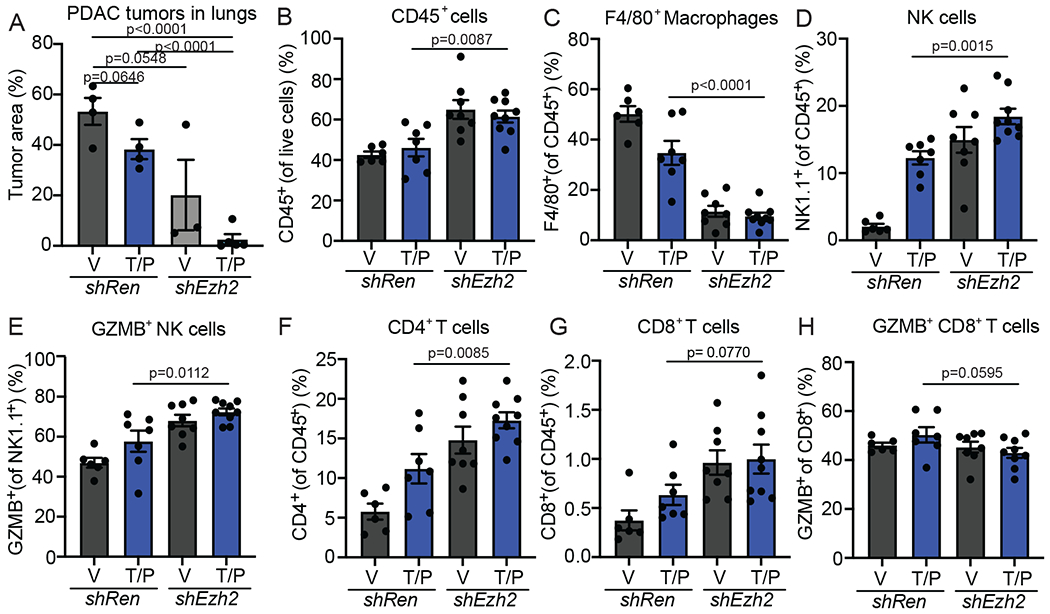
Combined EZH2 knockdown and T/P treatment reduces pancreatic metastasis growth and enhances NK and T cell immune surveillance in the lung. **a,** *KPC1* PDAC cells harboring *shRen* or *shEzh2* shRNAs were injected i.v. into 8-12 week old C57BL/6 female mice. Following tumor formation in the lungs, mice were treated with vehicle (V) or combined trametinib (1mg/kg body weight) and palbociclib (100 mg/kg body weight) (T/P) for 2 weeks. Quantification of lung tumor burden after 2 weeks of treatment is shown (**a,** shRen V, n=4; shRen TP, n=4; shEzh2 V, n=3; shEzh2 TP, n=5 independent mice). **b-h,** Flow cytometry analysis of total CD45^+^ immune cells, F4/80^+^ macrophages, NK cells, T cells, and their expression of GZMB in *shRen* or *shEzh2 KPC1* PDAC tumors in the lung following treatment as in (**a**) (**b-h,** shRen V, n=6; shRen TP, n=7; shEzh2 V, n=8; shEzh2 TP, n=8 independent mice). *P* values in **a-g** were calculated using two-tailed, unpaired Student’s t-test. Error bars, mean ± SEM.

Combinatorial EZH2 knockdown and T/P treatment also had profound anti-tumor effects. Whereas T/P treatment in the control *shRen* setting or EZH2 knockdown alone led to some reduction in tumor growth, T/P treatment in the context of EZH2 knockdown produced significant tumor control, with many tumors regressing after just two-week treatment (Fig. 5f,g and Extended Data Fig. 7g). Remarkably, the majority of *shEzh2 KPC1*-derived tumors treated with T/P continued to regress and completely responded (Fig. 5g–i). Indeed, while mice harboring control *shRen* PDAC treated with vehicle or T/P or *shEzh2* PDAC treated with vehicle quickly succumbed to the disease, 11/15 mice harboring *shEzh2* tumors treated with T/P had complete responses that remained durable even after treatment ceased (Fig. 5h,i). *KPC2* PDAC transplant mice also showed enhanced survival following EZH2 knockdown and treatment with T/P, albeit to a lesser extent with 2/8 complete responders (Extended Data Fig. 7h). Combined EZH2 knockdown and T/P treatment also synergized to reduce tumor growth and further activate NK and T cell immune responses in established PDAC lung metastases (Extended Data Fig. 8).

Some PDAC-bearing mice were treated with NK1.1 (PK136) or CD8 (2.43) depleting monoclonal antibodies (mAbs) simultaneously with drug administration to assess whether activation of NK and/or CD8^+^ T cell immunity was responsible for tumor control. Strikingly, both NK or CD8^+^ T cell depletion mitigated long-term survival and prevented complete tumor responses induced following T/P treatment of animals with EZH2 suppressed *KPC1* and *KPC2* PDAC tumors (Fig. 5h,i and Extended Data Fig. 7h). Together these findings demonstrate that EZH2 knockdown can potentiate senescence-mediated long-term tumor control in PDAC through mobilization of cytotoxic NK and T lymphocyte immunity.

### EZH2 suppression reinstates chemokines for lymphocyte trafficking

**Fig. 6.**
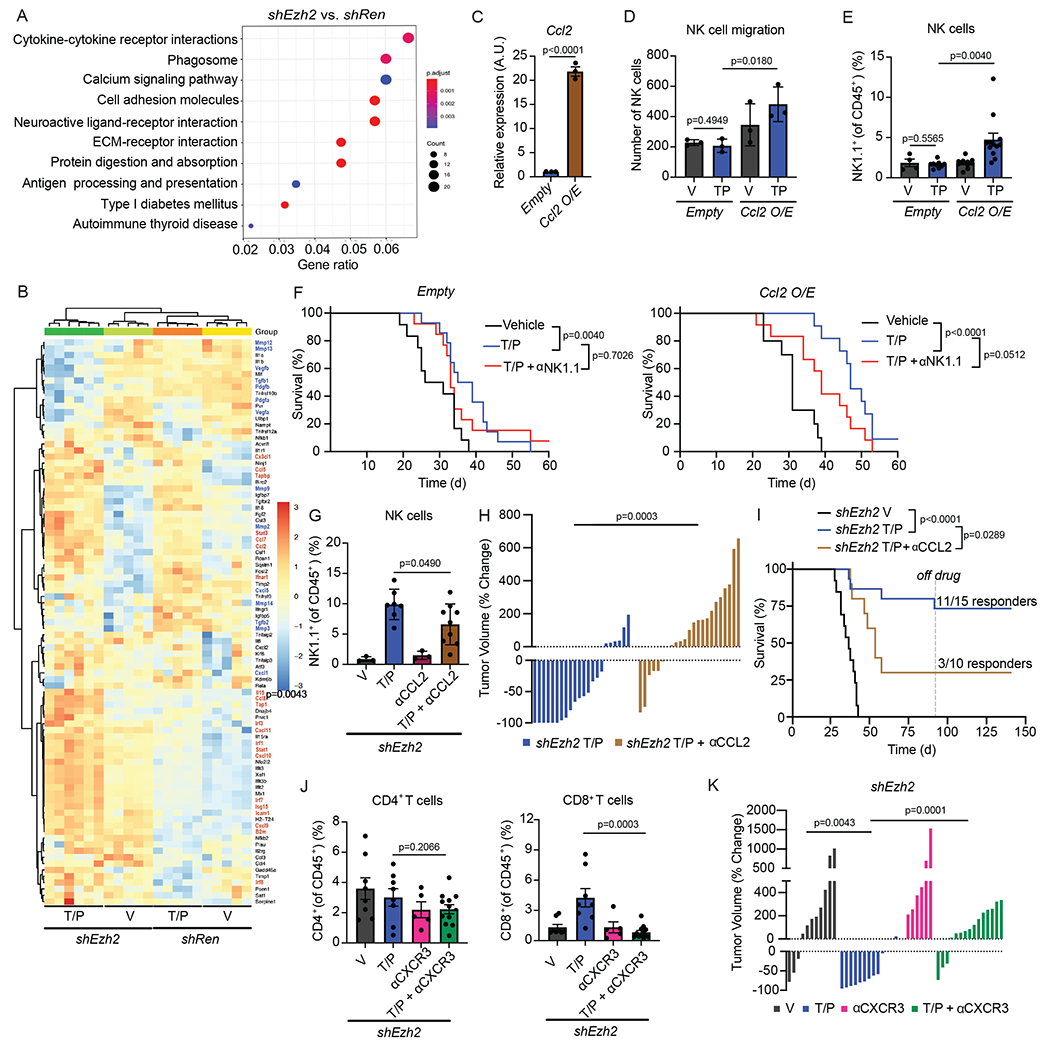
EZH2 suppression reinstates SASP-associated chemokines to drive NK and T cell accumulation in PDAC. **a**, KEGG pathway analysis of RNA-seq data showing enriched pathways in *shEzh2* compared to *shRen KPC*1 orthotopic PDAC tumor cells FACS sorted from C57BL/6 female mice treated with trametinib (1mg/kg) and palbociclib (100mg/kg) for 2 weeks (n=5-6 per group). **b**, Heatmap of RNA-seq analysis of SASP gene expression in tumor cells FACS sorted from *KPC*1 orthotopic PDAC tumors harboring indicated shRNAs and treated as in (**a**) (n=5-6 per group). **c**, qRT-PCR analysis of *Ccl2* expression in *KPC1* PDAC cells engineered to overexpress (O/E) a *Ccl2* cDNA or *Empty* control vector (n=3 per group). A.U., arbitrary units. **d**, NK cell migration assay in the presence of conditioned media from *KPC1* PDAC cells engineered to overexpress *Ccl2* or *Empty* vector and treated with vehicle or trametinib (25nM) and palbociclib (500nM) for 8 days (n=3 per group). **e**, Flow cytometry analysis of NK cell numbers in *KPC*1 orthotopic PDAC tumors expressing control *Empty* or *Ccl2* vectors following treatment as in (**b**) (**e,** Empty V, n=4; Empty TP, n=9; CCL2 O/E, n=11 and CCL2 O/E/TP, n=12 independent mice). Data represents pool of 3 independent experiments. **f**, Kaplan-Meier survival curve of mice with *KPC*1 orthotopic PDAC tumors expressing control *Empty* (left) or *Ccl2* (right) vectors treated with vehicle, combined trametinib (1mg/kg) and palbociclib (100mg/kg), and/or an NK1.1 depleting antibody (PK136; 250 μg) (**f,** Empty V, n=12; Empty TP, n=14; Empty TP+αNK1.1, n=13; CCL2O/E V, n=10; CCL2 O/E TP, n=11 and CCL2 O/E TP+αNK1.1, n=12 independent mice). Data represents pool of 2 independent experiments. **g**, Flow cytometry analysis of NK cell numbers in *shEzh2 KPC*1 orthotopic PDAC tumors following treatment with vehicle, combined trametinib (1mg/kg) and palbociclib (100mg/kg), and/or a CCL2 depleting antibody (2H5; 200 μg) for 2 weeks (**g,** V, n=3; αCCL2, n=3; TP, n=7 and TP αCCL2, n=9 independent mice). Data represents pool of 2 independent experiments. **h**, Waterfall plot of the response of *shEzh2 KPC1* orthotopic PDAC tumors to treatment as in (**g**) (**h,** shEzh2 TP, n=22 and shEzh2 TP αCCL2, n=23 independent mice). Data represents pool of 3 independent experiments. **i**, Kaplan-Meier survival curve of mice with *shEzh2 KPC*1 orthotopic PDAC tumors treated as in (**g**) (**i,** shEzh2 V, n=13; shEzh2 TP, n=15 and shEzh2 TP αCCL2, n=10 independent mice) Values for *shEzh2* V and T/P treated cohorts are the same displayed in Figs. 5h and 5i. Dotted line indicates when mice were taken off of treatment. Data represents pool of 2 independent experiments. **j**, Flow cytometry analysis of CD4^+^ and CD8^+^ T cell numbers in *shEzh2 KPC*1 orthotopic PDAC tumors following treatment with vehicle, combined trametinib (1mg/kg) and palbociclib (100mg/kg), and/or a CXCR3 depleting antibody (CXCR3-173; 200 μg) for 2 weeks (**j,** V, n=8; αCXCR3, n=5; TP, n=8; and TP+αCXCR3, n=12 independent mice). Data represents pool of 2 independent experiments. **k**, Waterfall plot of the response of *shEzh2 KPC1* orthotopic PDAC tumors to treatment as in (**j**) (**k,** V, n=11; αCXCR3, n=7; TP, n=13; and TP+αCXCR3, n=15 independent mice). Data represents pool of 2 independent experiments. *P* values in **a** were calculated using two-sided, hypergeometric test, **c,d,e,g,h,j,k** using two-tailed, unpaired Student’s t-test, and **f** and **i** using log-rank test. Error bars, mean ± SEM.

Given the numerous cell autonomous and non-cell autonomous functions of EZH2 in cancer biology^48^, we performed bulk RNA-seq on FACS sorted GFP^+^ tumor cells isolated from drug-treated *shRen* or *shEzh2* PDAC tumors to understand the mechanisms by which EZH2 targeting led to immune-mediated tumor control (Supplementary Table 40). Unbiased KEGG pathway analysis revealed “cytokine-cytokine receptor interaction” and “cell adhesion molecules” as top differentially regulated pathways when comparing *shEzh2* vs. *shRen* tumors treated with T/P (Fig. 6a and Supplementary Tables 41–45). Further analysis uncovered significantly increased expression of SASP-associated pro-inflammatory cytokines and chemokines (*Il15, Ccl2/5/7/8, Cxcl9/10/11, Cx3cl1)*, as well as genes involved in antigen presentation/processing (*B2m, Tap1, Tapbp*) and cell adhesion (*Icam1*) important for T and NK cell recognition of tumor cells in the context of T/P-induced senescence and EZH2 suppression (Fig. 6b and Supplementary Table 46).

**Extended Data Fig. 9.**
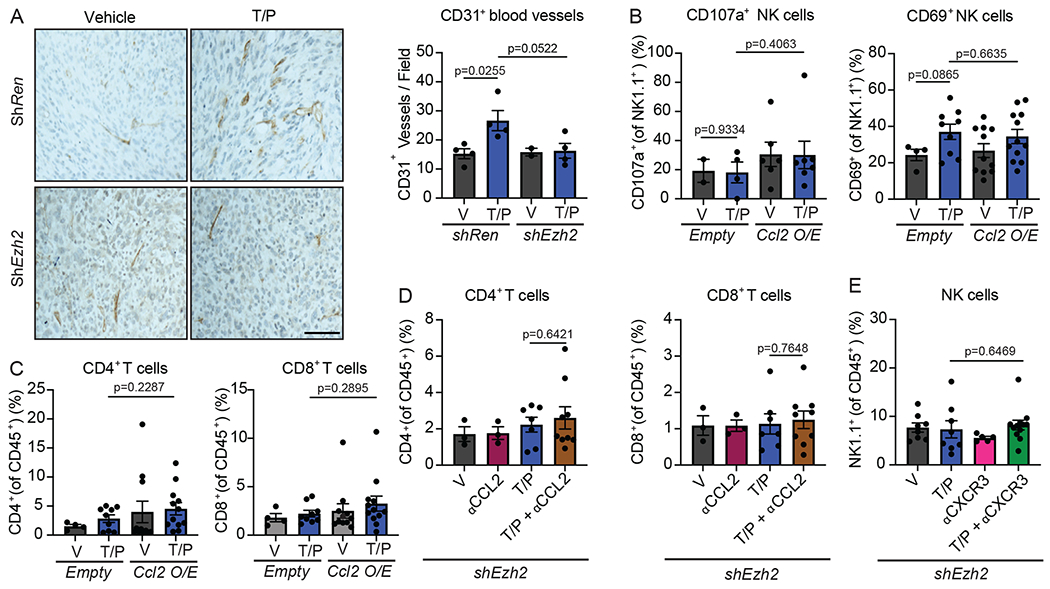
EZH2 blockade reduces T/P-induced blood vessel formation and promotes CCL2 and CXCL9/10 secretion that increases NK and CD8^+^ T cell infiltration into PDAC. **a**, IHC staining of *KPC1* orthotopic PDAC tumors harboring *shRen* or *shEzh2* shRNAs treated with vehicle or combined trametinib (1mg/kg) and palbociclib (100 mg/kg) (T/P) for 2 weeks. Quantification of blood vessels per field are shown on inset (**a,** shRen V, n=4; shRen TP, n=4; shEzh2 V, n=2; shEzh2 TP, n=4 independent tumors). Scale bar, 50μm. **b-c**, Flow cytometry analysis of NK cell activation markers (**b**) and CD4^+^ and CD8^+^ T cell numbers (**c**) in *KPC1* orthotopic PDAC tumors expressing control *Empty* or *Ccl2* vectors and treated as in (**a**) (**b-c,** Empty V, n=4; Empty TP, n=9; Empty CCL2O/E V, n=11; CCL2 O/E TP, n=12 independent mice). Data represents pool of 3 independent experiments **d**, Flow cytometry analysis of CD4^+^ and CD8^+^ T cell numbers in *shEzh2 KPC1* orthotopic PDAC tumors following treatment with vehicle, combined trametinib (1mg/kg) and palbociclib (100mg/kg), and/or a CCL2 depleting antibody (2H5; 200 μg) for 2 weeks (**d,** shEzh2 V, n=3; shEzh2 αCCL2, n=3; shEzh2 TP, n=7; shEzh2 TP+αCCL2, n=9 independent mice). Data represents pool of 2 independent experiments. **e**, Flow cytometry analysis of NK cell numbers in *shEzh2 KPC1* orthotopic PDAC tumors following treatment with vehicle, combined trametinib (1mg/kg) and palbociclib (100mg/kg), and/or a CXCR3 depleting antibody (CXCR3-173; 200 μg) for 2 weeks (**d,** V, n=8; TP, n=8; αCCXR3, n=5; TP+αCCXR3, n=12 independent mice). Data represents pool of 2 independent experiments. *P* values in **a-e** were calculated using two-tailed, unpaired Student’s t-test. Error bars, mean ± SEM.

The expression of other SASP-associated factors showed differential responses to EZH2 blockade. Whereas transcriptional regulators of the pro-inflammatory SASP that are normally repressed in the PDAC TME, including STING (*Irf1/3/7/8, Ifnar, Isg15*) and STAT (*Stat1/3*) pathway components, were upregulated following therapy-induced senescence and EZH2 suppression, many of the pro-angiogenic (*Vegfa/b, Pdgfa/b, Mmp3/9/12/13/14*) and immune suppressive (*Tgfb1/2, Cxcl1/5*) SASP factors normally induced during senescence were downregulated (Fig. 6b and Supplementary Table 46). Indeed, the increase in blood vessels observed following T/P treatment in *shRen* tumors and as reported in our previous study^20^ was not found in s*hEzh2* tumors (Extended Data Fig. 9a). Thus, EZH2 suppression following T/P-induced senescence triggers a phenotypic switch in the SASP program in the PDAC TME from a pro-angiogenic SASP to a pro-inflammatory SASP that may contribute to enhanced cytotoxic lymphocyte anti-tumor immunity.

Many of the most highly induced pro-inflammatory SASP factors upon combined EZH2 knockdown and therapy-induced senescence in the PDAC TME are chemokines known to attract NK and T cells from the periphery into inflamed tissues^33^, including CCL2 and CXCL9/10. In addition to its impact on monocyte and macrophage trafficking, CCL2 can also attract NK cells expressing its receptor CCR2^49^, as has been previously shown in senescent liver cancer lesions^50^. To interrogate the role of CCL2 in anti-tumor NK cell responses in PDAC, we first engineered *KPC* PDAC cell lines to express a *Ccl2* cDNA (or an *Empty* vector as a control) (Fig. 6c). *In vitro*, conditioned media from *KPC1* cells overexpressing CCL2 and pre-treated with T/P produced significantly more NK cell migration through a transwell insert (Fig. 6d), demonstrating that CCL2 secretion by senescent tumor cells can facilitate NK cell chemotaxis. *Empty* or *Ccl2* expressing *KPC* cells were then transplanted orthotopically into the pancreas of C57BL/6 mice and animals randomized into treatment groups following tumor formation to assess the impact on NK cell immune surveillance *in vivo*. Flow cytometry analysis revealed that tumor-specific CCL2 overexpression in the context of T/P-induced senescence was sufficient to significantly increase NK cell accumulation into PDAC without affecting NK cell activation (Fig. 6e and Extended Data Fig. 9b). This influx of NK cells into CCL2 overexpressing tumors prolonged the survival of PDAC-bearing animals treated with T/P, as NK cell depletion with an NK1.1-targeting mAb significantly diminished the survival benefit (Fig. 6f).

To determine whether EZH2 suppression facilitated anti-tumor NK cell immunity through CCL2 induction, we also treated mice transplanted with *shEzh2 KPC1* PDAC tumors with vehicle, T/P, and/ or a mAb targeting CCL2 (2H5). Indeed, CCL2 was required for these anti-tumor immune effects, as CCL2 blockade reduced NK cell accumulation, abolished tumor regressions, and significantly mitigated the survival benefit and number of complete responders to combined EZH2 knockdown and T/P treatment (Fig. 6g–i). Thus, SASP-associated CCL2 is both necessary and sufficient to drive NK cell infiltration and potentiate NK cell-mediated tumor control in PDAC following T/P-induced senescence.

In contrast to its effects on NK cells, CCL2 overexpression or neutralization had little impact on CD4^+^ and CD8^+^ T cell recruitment (Extended Data Fig. 9c,d), suggesting other SASP chemokines may influence T cell chemotaxis. T cells express the receptor CXCR3 that binds chemokines CXCL9/10/11 that are critical for CD8^+^ T cell homing to the TME and ICB immunotherapy efficacy^51–54^. In agreement, treatment of *shEzh2* PDAC-bearing mice with a CXCR3 mAb (CXCR3-173) blunted CD8^+^ and to a lesser extent CD4^+^ T cell accumulation without affecting NK cell numbers following T/P treatment (Fig. 6j and Extended Data Fig. 9e). This reduction in CD8^+^ T cell recruitment upon CXCR3 blockade also mitigated the anti-tumor effects of combined EZH2 suppression and T/P-induced senescence and reversed PDAC tumor regressions (Fig. 6k). Therefore, distinct SASP chemokines are important for NK and T cell migration into the PDAC TME and enable the immune-mediated anti-tumor effects observed upon EZH2 suppression.

### Combined Tazemetostat and T/P treatment activates anti-tumor immunity in preclinical models

**Fig. 7.**
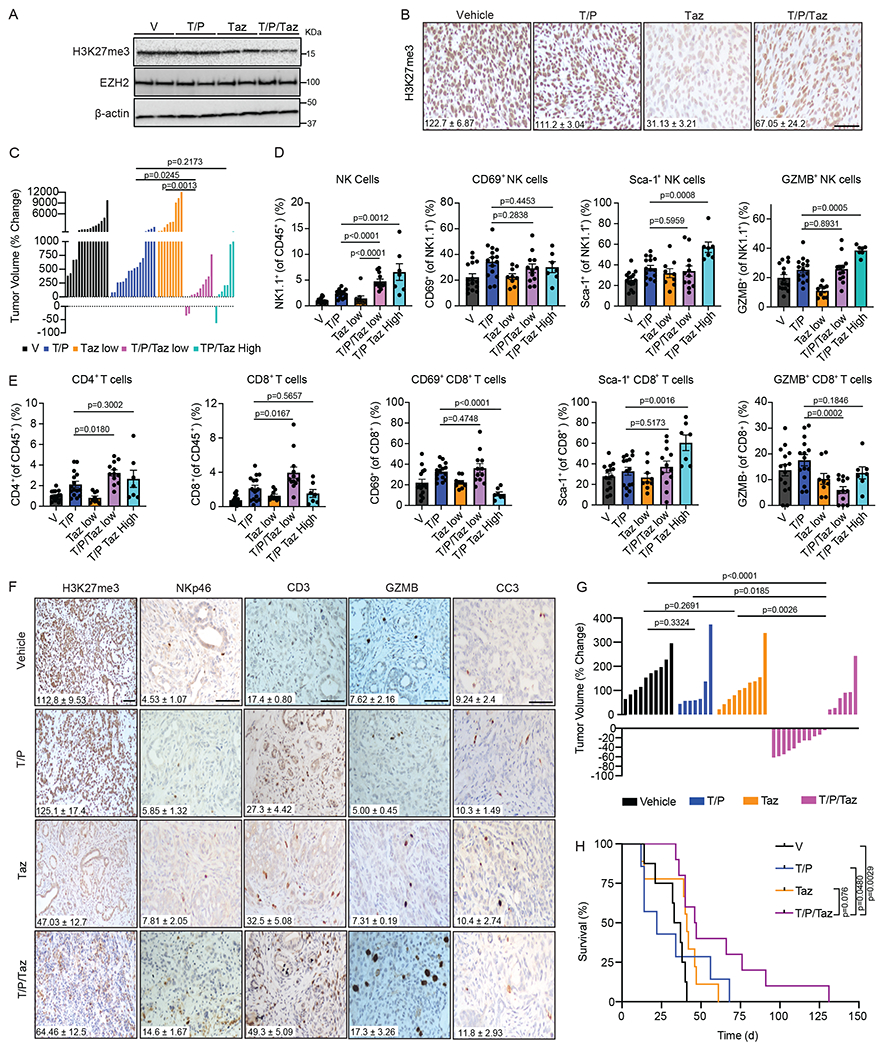
Pharmacological EZH2 methyltransferase inhibition in combination with T/P reactivates cytotoxic NK and T cell immunity and enhances tumor control in preclinical PDAC models. **a**, Immunoblots of *KPC1* orthotopic PDAC tumors from C57BL/6 female mice treated with vehicle, trametinib (1 mg/kg) and palbociclib (100 mg/kg), and/or tazemetostat (Taz) (125 mg/kg) for 2 weeks. **b**, Immunohistochemical (IHC) staining of *KPC1* orthotopic PDAC tumors treated as in (**a**). H-score quantification of H3K27me3 expression is shown inset (n=3 per group). Scale bar, 50μm. **c**, Waterfall plot of the response of *KPC1* orthotopic PDAC tumors following 2 week-treatment with vehicle, trametinib (1 mg/kg) and palbociclib (100 mg/kg), and/or low (125 mg/kg) or high (400 mg/kg) doses of tazemetostat (**c,** V, n=15; Taz low, n=16; TP, n=9; TP/TAZ low, n=10 and TP/TAZ high, n=7 independent mice). Data represents pool of 3 independent experiments. **d-e**, Flow cytometry analysis of NK (**d**) and T cell (**e**) numbers and activation markers in *KPC1* orthotopic PDAC tumors following treatment as in (**c**) (**d-e,** V, n=15; Taz low, n=15; TP, n=9; TP/TAZ low, n=12 and TP/TAZ high, n=7 independent mice). Data represents pool of 3 independent experiments. **f**, IHC staining of *KPC* GEMM tumors treated as in (**a**). Quantification of the number of NKp46^+^ NK cells, CD3^+^ T cells, and GZMB^+^ and Cleaved Caspase-3 (CC3)^+^ cells per field, and H-scores for H3K27me3 expression, are shown inset (**f,** V; TP; TAZ, n=3 and TP/TAZ, n=3 independent tumors). Scale bars, 50μm. **g**, Waterfall plot of the response of *KPC* GEMM tumors to treatment as in (**a**) (**g,** V, n=10; TP, n=7; TAZ, n=10 and TP/TAZ, n=17 independent mice). **h,** Kaplan-Meier survival curve of *KPC* GEMM male and female mice treated as in **(a)** (**h,** V, n=8; TP, n=7; TAZ, n=9 and TP/TAZ, n=10 independent mice). *P* values in **c-e** and **g** were calculated using two-tailed, unpaired Student’s t-test, and those in **h** calculated using log-rank test. Error bars, mean ± SEM.

Given the profound anti-tumor effects of genetic EZH2 knockdown in combination with therapy-induced senescence in PDAC, we next tested whether small molecule EZH2 inhibitors that are in clinical development could achieve similar responses. Mice transplanted orthotopically with *KPC1* PDAC cells were randomized and treated with vehicle, T/P, and/or the FDA-approved EZH2 methyltransferase inhibitor tazemetostat (Taz). Two-week treatment with Taz effectively decreased H3K27me3 levels and, in combination with T/P, significantly reduced tumor growth compared to either treatment arm alone (Fig. 7a–c). Combined Taz and T/P treatment also led to a significant increase in NK cell numbers and their expression of activation (e.g. Sca-1) and cytotoxicity (e.g. GZMB) markers (Fig. 7d). Interestingly, whereas CD4^+^ and CD8^+^ T cell numbers increased at lower doses of Taz (125 mg/kg) in combination with T/P, high Taz concentrations (400 mg/kg) reduced CD8^+^ T cell numbers and expression of CD69, a marker of early activation and proliferation (Fig. 7e). This suggests that, separate from its action on tumor cells, Taz at high concentrations may affect T cell proliferation in a manner that reduces its anti-tumor activity. We therefore performed the remaining studies using the lower 125 mg/kg Taz dose.

To test this inhibitor combination in an autochthonous model, we utilized *P48-Cre*; *Kras^LSL-G12D/wt^*;*Trp53^fl/wt^* (*KPC*) GEMM mice that spontaneously develop PDAC that closely resembles the human disease. Whereas T/P or Taz treatment alone increased T cell infiltration into PDAC tumors, single agent treatment did not cause NK cell mobilization or enhanced immune cytotoxicity (as marked by GZMB expression), and resulted in only marginal changes in tumor growth and overall survival of *KPC* GEMM animals (Fig. 7f–h). In contrast, combined T/P and Taz treatment led to further increased T cell and NK cell infiltration and GZMB expression, culminating in tumor regressions in 8/17 of PDAC-bearing KPC GEMMs and significantly increased overall survival in the absence of a detectable apoptotic response (Fig. 7f–h). Thus, both genetic EZH2 suppression and EZH2 methyltransferase inhibitor treatment can augment T/P-induced senescence to potentiate cytotoxic NK and T cell immunity and tumor control in transplanted and autochthonous PDAC models.

### EZH2 associated with NK and T cell immune suppression and poor survival in PDAC patients

**Fig. 8.**
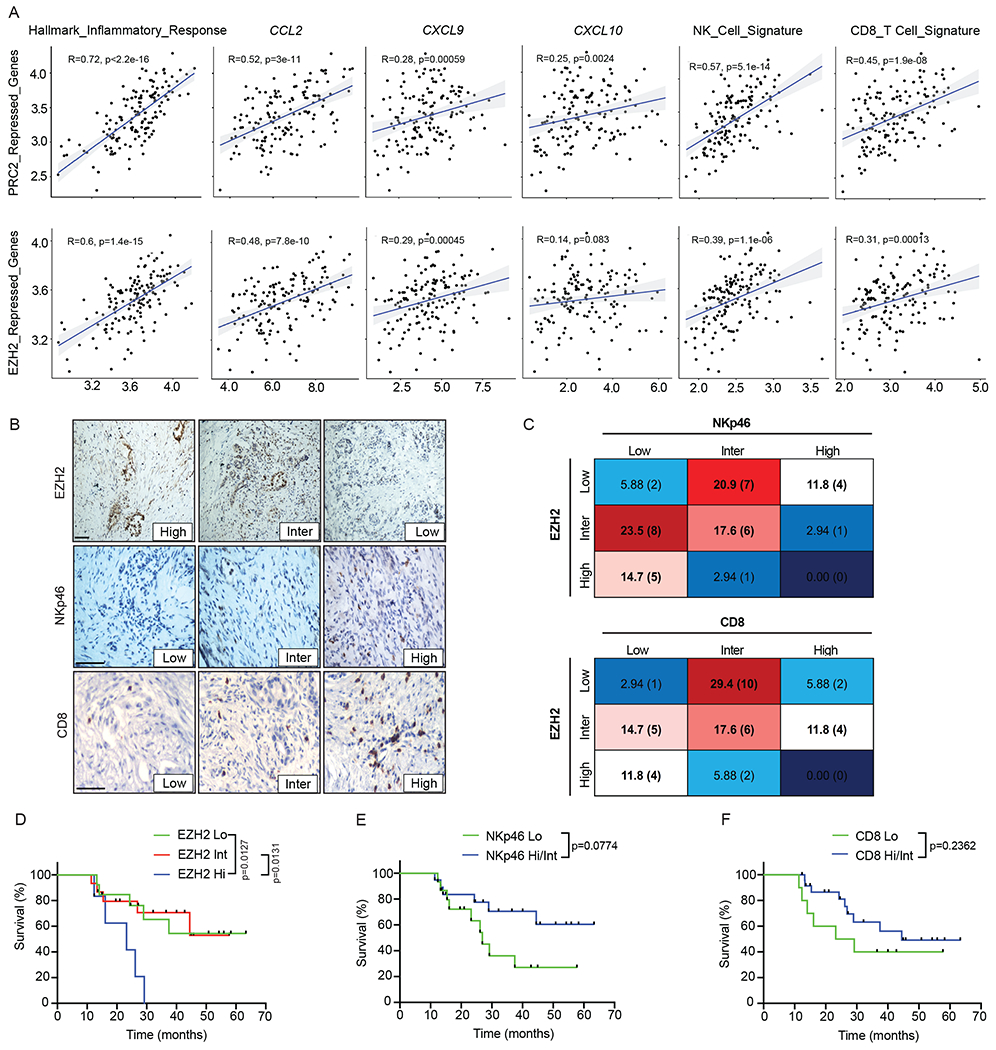
EZH2 is associated with suppression of inflammatory chemokine signaling, reduced NK and T cell immune surveillance, and poor survival in PDAC patients. **a**, Pearson’s correlation analysis plots comparing signatures of EZH2 and PRC2 repressed genes with inflammatory response gene sets, *CCL2*, *CXCL9*, and *CXCL10* expression, and NK and CD8^+^ T cell signatures in human primary PDAC transcriptomic data^55^ (n=145 samples). Line represents line of best fit. **b**, Representative IHC staining of surgically resected human PDAC tumors (n=34). Scale bars, 50μm. **c**, Scoring of EZH2, NKp46, and CD8 expression from IHC staining in (b) (n=34). Percentage of samples with indicated scores are shown, with the total number of samples in parentheses. **d**, Kaplan-Meier survival curve of human PDAC patients stratified based on EZH2 expression levels in (**b**) (n=13, 15, and 6 for EZH2 Lo, Int, and Hi, respectively). **e**, Kaplan-Meier survival curve of human PDAC patients stratified based on NKp46 expression levels in (**b**) (n=15 and 19 for NKp46 Lo and Hi/Int, respectively)*.* **f**, Kaplan-Meier survival curve of human PDAC patients stratified based on CD8 expression levels in (**b**) (n=10 and 24 for CD8 Lo and Hi/Int, respectively)*. P* values in **a** were calculated using two-tailed, unpaired Student’s t-test, and those in **d-f** were calculated using log-rank test. Error bars, mean ± SEM.

Finally, we set out to evaluate the relationship between EZH2 activity, inflammatory signaling, and NK and T cell immunity in human PDAC. We first interrogated a previously published gene expression dataset containing 145 PDAC patient samples^55^. Expression of EZH2 and PRC2 repressed genes correlated positively with inflammatory response genes, including *CCL2, CXCL9*, and *CXCL10* that are important of NK and T cell trafficking into the PDAC TME (Fig. 8a and Supplementary Table 47). Moreover, NK and CD8^+^ T cell gene transcript levels were also significantly associated with expression of EZH2 repressed genes (Fig. 8a and Supplementary Table 47). These results support a relationship between EZH2 activity, inflammatory chemokine signaling, and cytotoxic lymphocyte infiltration in human PDAC.

We then performed immunohistochemical (IHC) staining and blinded scoring on formalin-fixed, paraffin-embedded (FFPE) surgically resected tumor specimens from PDAC patients treated at UMass Memorial hospital to assess the relationship between EZH2 expression, NKp46^+^ NK cells and CD8^+^ T cell numbers, and patient survival. The 34 patient samples analyzed presented a spectrum of EZH2 expression and NK and CD8^+^ T cell densities, with high/intermediate EZH2 levels correlating with low NK and CD8^+^ cell numbers and low EZH2 expression with high/intermediate lymphocyte penetrance (Fig. 8b,c). Patients with high EZH2 expression in their primary PDAC lesions had significantly reduced overall survival (Fig. 8d). In contrast, high/intermediate intratumoral NK and CD8^+^ cell numbers showed a trend towards improved patient survival, although the effect did not achieve significance (Fig. 8e,f). Taken together, our work demonstrates that EZH2 is associated with suppression of inflammatory signaling, NK and T cell dysfunction, and reduced survival in murine and human PDAC, and that targeting EZH2 activity can restore long-term innate and adaptive immune-mediated PDAC control.

## DISCUSSION

PDAC remains without durable chemo-, targeted, and immunotherapy regimens, and as such has a dismal 5-year survival rate of 11%^1^. Many promising studies and clinical trials have focused on overcoming immune suppression as a therapeutic strategy in PDAC through (a) re-engineering T cell responses via CAR-T, ICB therapy, or neo-antigen vaccine approaches, (b) targeting suppressive fibroblast populations and functions, and/or (c) eliminating or reprogramming suppressive myeloid cells^56,57^. Here we investigated how to remodel the tumor secretome directly as a strategy to enhance tumor immunogenicity and transform the immune suppressive PDAC TME. By comparing the effects of therapy-induced senescence in KRAS mutant LUAD and PDAC tumors, we uncovered an epigenetic mechanism that suppresses the pro-inflammatory SASP secretome in PDAC that is mediated by PRC2 component EZH2 and its methyltransferase activity. EZH2 inhibition in combination with therapy-induced senescence unleashes pro-inflammatory SASP chemokines such as CCL2 and CXCL9/10 and induces MHC-I and NK ligand expression to orchestrate an innate and adaptive immune attack through cytotoxic NK and T lymphocytes that in some cases led to complete responses in preclinical PDAC models.

EZH2 has been shown to facilitate tumor immune evasion and resistance to ICB therapy in other cancer settings^58–61^. Here we uncovered a mechanism by which EZH2 mediates PDAC immune suppression through inhibition of the pro-inflammatory transcriptome, secretome, and surfaceome associated with the SASP. Mechanistically, EZH2 methyltransferase activity was directly responsible for suppressing many SASP-related chemokine and cytokine genes, such that genetic or pharmacological inhibition of EZH2 in tumor cells triggered to senescence following T/P therapy led to a marked reduction in H3K37me3 levels at these loci and a phenotypic switch from a pro-angiogenic to a pro-inflammatory SASP program. Though not explored in our current study, other outcomes of EZH2 protein suppression, including activation of DNA damage response (DDR) pathways and/or cGAS/STING components, could also indirectly contribute to SASP factor expression independent of EZH2 methyltransferase activity in PDAC^42,62,63^. Clinically, EZH2 is commonly overexpressed in poorly differentiated PDAC and associated with chemoresistance^64,65^. Our analysis of patient samples in addition reveals that EZH2 is not only associated with suppression of inflammatory chemokines and NK and T cell immunity in the human disease, but also poor overall patient survival. Thus, our work identifies EZH2 as an important marker and inducer of immune suppression in PDAC that is therapeutically targetable.

The SASP is often considered a “double-edged sword” and can promote anti-tumor immune surveillance or alternatively pro-tumor immune evasion depending on the context^21,22,28^. Here we find that the resident tissue context plays a key role in immune responses to senescence stimuli, with immune-stimulatory SASP cytokines and chemokines and subsequent NK cell surveillance potentiated in the lung but not the pancreas TME. Though technical differences in how tumor cells were transplanted into the lung (i.v. injection) and pancreas (direct injection of bolus of cells) could impact NK cell responses, pancreas tumor cells injected as a bolus directly into the liver were able to elicit NK cell anti-tumor immunity following T/P treatment, suggesting the pancreas TME per se promotes resistance to senescence-driven NK cell surveillance. Indeed, we found that SMA^+^ myofibroblasts that are abundant in the PDAC TME contribute to NK as well as T cell immune suppression by promoting EZH2-mediated repression of pro-inflammatory SASP genes in tumor cells. Of note, unlike LUAD cells that were null for p53, transplanted PDAC cells contained a mutant p53*^R172H^* allele that could exert additional immune suppressive phenotypes^66^, and as such we cannot rule out the possibility that tumor cell intrinsic factors may also contribute to differences in inflammatory signaling pathways following senescence induction^67^. Nonetheless, we believe our study demonstrates that the quantity and quality of the SASP elicited following senescence induction is influenced in part by the TME and its impact on the epigenetic state of the cancer cell.

Our findings suggest that induction of chemokines through the SASP that drive NK and T cell trafficking into TMEs could be a powerful approach to make immunologically “cold” tumors such as PDAC “hot”^68^. While often considered a monocyte chemoattract, our results support the emerging view that CCL2 is also an important stimulator of NK cell chemotaxis into senescent tumors^50^. Chemokines CCL7 and CCL8 are also highly induced in senescent PDAC cells following EZH2 inhibition and bind to the same receptor as CCL2 (CCR2), suggesting they could also play a role in NK cell trafficking into PDAC. Other SASP chemokines such as CXCL9/10 that bind to CXCR3 and are associated with T cell recruitment and “hot” TMEs in other cancer settings^51,52,54,69^ are necessary for CD8^+^ T cell recruitment into PDAC following therapy-induced senescence. Remarkably, in our system, combining increased cytotoxic NK and CD8^+^ T cell trafficking via SASP chemokines with the enhanced immunogenicity of senescent cells is sufficient to potentiate anti-tumor immune surveillance in PDAC even in the absence of immune checkpoint blockade.

Though both genetic EZH2 suppression and pharmacological EZH2 methyltransferase inhibition lead to NK and T cell activation and enhanced PDAC tumor control following therapy-induced senescence, the anti-tumor effects of small molecule EZH2 inhibition are not as robust or durable as compared with its genetic knockdown. The deleterious effects of systemic EZH2 inhibition on CD8^+^ T cell function and proliferation^70^, as well as the scaffolding functions of EZH2 acting independently of its methyltransferase activity^62,63^, may contribute to the reduced anti-tumor activity of Tazemetostat. Inhibitors that disrupt PRC2 complex stability, for instance by targeting the core subunit EED^71,72^, or EZH2 degraders^73–75^, may offer more potent PRC2 complex and EZH2 suppression.

Tazemetostat and other EZH2 methyltransferase inhibitors have demonstrated efficacy and been implemented into the clinical care of hematological malignancies and sarcomas; however, they have yet to show potent activity as single agents in solid tumors^76^. Our findings provide rationale for combining EZH2 inhibitors with a senescence-inducing therapy – here produced by a MEK and CDK4/6 inhibitor combination – to promote NK and T cell-mediated eradication of senescent PDAC lesions through pro-inflammatory SASP induction. As radiation and chemotherapy can also induce senescence in some settings, it will be interesting to see whether EZH2 inhibitors show combinatorial activity with these agents as well. Collectively, our work provides a strategy for leveraging EZH2 inhibitors as an immune oncology approach in combination with senescence-inducing agents to remodel the inflammatory tumor secretome for immune-mediated PDAC control.

## METHODS

### Ethical regulations

The research performed in this study complies with all ethical regulations. All mouse experiments were approved by the University of Massachusetts Chan Medical School Internal Animal Care and Use Committee (IACUC) (PROTO202000077). Surgically resected PDAC patient samples were acquired under the University of Massachusetts Chan Medical School IRB protocol no. H-4721. Informed consent was obtained from all participants.

### Cell lines and compounds

PANC-1 (CRL-1469) and 293T (CRL-3216) cells were purchased from the American Type Culture Collection (ATCC). Murine PDAC (*KPC1, KPC2*) and LUAD (*KP1, KP2*) cell lines were generated as previously described^19,20^. For visualizing and tracking *KPC* and *KP* tumor cell lines with luciferase and GFP *in vivo,* cells were transduced with the following retroviral constructs: MSCV-luciferase (luc)-IRES-GFP (for *KPC1* and *KP1*), MSCV-IRES-GFP (for *KP2*), and MSCV-shRen-PGK-Puro-IRES-GFP (for *KPC2*). Retroviruses were packaged by co-transfection of Gag-Pol expressing 293T cells with expression constructs and envelope vectors (VSV-G). Following transduction, cells were purified by FACS sorting the GFP^+^ population on a FACSAria (BD Biosciences). All cells were maintained in a humidified incubator at 37°C with 5% CO_2_, and grown in DMEM supplemented with 10% FBS and 100 IU/ml penicillin/streptomycin (P/S). *KPC* cell lines were grown in culture dishes coated with 100 μg/ml collagen (PureCol) (5005; Advanced Biomatrix). All cell lines used were negative for mycoplasma. Human cell lines were authenticated by their source repository.

Trametinib (S2673), palbociclib (S1116), and GSK126 (S7061) were purchased from Selleck chemicals and tazemetostat (HY-13803) from MedChemExpress for *in vitro* studies. Drugs for *in vitro* studies were dissolved in DMSO (vehicle) to yield 10mM stock solutions and stored at −80°C. For *in vitro* studies, growth media with or without drugs was changed every 2-3 days. For *in vivo* studies, trametinib (T-8123) and palbociclib (P-7744) were purchased from LC Laboratories, tazemetostat (HY-13803) purchased from MedChemExpress, and ganciclovir (GCV) purchased from Invivogen. Trametinib was dissolved in a 0.5% hydroxypropyl methylcellulose and 0.2% Tween-80 solution, palbociclib in 50 mM sodium lactate buffer (pH 4), tazemetostat in a 0.5% sodium carboxymethylcellulose and 0.1% Tween-80 solution (Sigma-Aldrich), and GCV in PBS.

### Short-hairpin RNA (shRNA) knockdown

shRNAs targeting *Ezh2*, *Suz12*, and *Renilla (Ren)* were cloned into the XhoI EcoRI locus of MLP retroviral vectors (MSCV-LTR-shRNA-PGK-Puro-IRES-GFP) as previously described^77^. Retroviruses were packaged by co-transfection of Gag-Pol expressing 293T cells with expression constructs and envelope vectors (VSV-G) using polyethylenimine (PEI; Sigma-Aldrich). Following transduction with shRNA retroviral constructs, cell selection was performed with 4μg/ml puromycin for 1 week. Knockdown was confirmed by immunoblot and immunohistochemistry.

### CCL2 overexpression

Murine *Ccl2* cDNA was cloned into an MSCV-based retroviral vector (MSCV-blast). Retroviruses were packaged by co-transfection of Gag-Pol expressing 293T cells with expression constructs and envelope vectors (VSV-G) using polyethylenimine (PEI; Sigma-Aldrich). Following transduction with *Ccl2* or control *Empty* constructs, cell selection was performed with 10μg/ml Blasticidin S for 1 week. *Ccl2* expression was confirmed by qRT-PCR.

### SA-β-gal staining

SA-β-gal staining was performed as previously described at pH 5.5 for mouse cells and tissue^19,20^. Fresh frozen sections of tumor tissue, or adherent cells plated in 6-well plates, were fixed with 0.5% glutaraldehyde in PBS for 15 min, washed with PBS supplemented with 1mM MgCl_2_, and stained for 4–18 hours in PBS containing 1 mM MgCl_2_, 1mg/ml X-Gal, and 5 mM each of potassium ferricyanide and potassium ferrocyanide. Tissue sections were counterstained with eosin. 5-10 high power 20x fields per tissue section were counted and averaged.

### Drug withdrawal clonogenic assays

*KPC* tumor cells were initially plated in 6-well plates and pre-treated with vehicle (DMSO), trametinib (25 nM), palbociclib (500 nM), and/or tazemetostat (5 μM) for 8 days. Pre-treated cells were then trypsinized, and 5×103 cells re-plated per well of a 6-well plate in the absence of drugs for 7 days. Remaining cells were fixed with methanol (1%) and formaldehyde (1%), stained with 0.5% crystal violet, and photographed using a digital scanner.

### Immunoblotting

Cell lysis was performed using RIPA buffer (Cell Signaling) supplemented with phosphatase inhibitors (5mM sodium fluoride, 1 mM sodium orthovanadate, 1 mM sodium pyrophosphate, 1 mM β-glycerophosphate) and protease inhibitors (Protease Inhibitor Cocktail Tablets, Roche). Protein concentration was determined using a Bradford Protein Assay kit (Biorad). Proteins were separated by SDS-PAGE and transferred to polyvinyl difluoride (PVDF) membranes (Millipore) according to standard protocols. Membranes were immunoblotted with antibodies (1:1,000) against EZH2 (5246), SUZ12 (3737), and H3K27me3 (9733) from Cell Signaling in 5% milk in TBS blocking buffer. After primary antibody incubation, membranes were probed with an ECL anti-rabbit IgG secondary antibody (1:10,000) from GE Healthcare Life Science and imaged using a ChemiDoc imaging system (BioRad). Protein loading was measured using a monoclonal β-actin antibody directly conjugated to horseradish peroxidase (A3854, Sigma-Aldrich; 1:20,000) and imaged as above.

### qRT-PCR

Total RNA was isolated using the RNeasy Mini Kit (Qiagen), and complementary DNA (cDNA) was generated using the TaqMan reverse transcription reagents (Applied Biosystems). qRT-PCR was performed in triplicate using SYBR Green PCR Master Mix (Applied Biosystems) on the StepOnePlus Real-Time PCR system (Applied Biosystems). β-actin or Gapdh served as endogenous normalization controls. qRT-PCR primer sequences can be found in Supplementary Table 48.

### Cytokine array

Cells were plated in duplicate or triplicate in 6-well plates and drug treated for 6 days. On day 6, 2 ml of new drug-containing media was added to each well and cells were incubated an additional 48 hours. Conditioned media was then collected and cells trypsinized and counted using a Countess II cell counter (Invitrogen). Media samples were then normalized based on cell number by diluting with culture media. Aliquots (75 μl) of the conditioned media were analyzed using a multiplex immunoassay (Mouse Cytokine/Chemokine 44-Plex array) from Eve Technologies.

### NK cell migration assay

Primary NK cells were isolated and enriched the day of the experiment from the spleens of 8-12 week old female C57BL/6 mice using the NK Cell Isolation Kit II according to manufacturer’s instructions (Miltenyi Biotec). 50,000 NK cells were then seeded in the top chamber of a transwell insert (Corning) in a 24-well dish in serum-Free DMEM media with 100 IU/ml penicillin/streptomycin. Serum-free conditioned media from *KPC* tumor cells (collected for 48 hrs following 6 day pre-treatment with indicated drugs) was then placed in the bottom chamber. Following 4 hr incubation in a 37°C cell culture incubator, NK cells migrating through the bottom chamber were fixed with 4% paraformaldehyde (PFA), stained with DAPI, and counted on a Celigo imaging cytometer (Nexcelom).

### Animal models

All mouse experiments were approved by the University of Massachusetts Chan Medical School Internal Animal Care and Use Committee (IACUC). Mice were maintained under specific pathogen-free conditions, and food and water were provided ad libitum. Housing conditions included a 12:12 light/dark cycle, with the lights coming on at 0700 and going off at 1900 daily, a temperature range of 68-79°F, and a humidity range of 30-70%. C57BL/6 mice were purchased from Charles River Laboratories and *P48-Cre* and *SMA-TK* strains purchased from Jackson Laboratory. *Trp53^fl/fl^* and *Kras^LSL-G12D/wt^* breeding pairs were generously provided by Wen Xue. For tumor transplantation studies into C57BL/6 mice, only female mice were used, as this greatly reduced costs and complications of housing adult male animals in the same cage. For studies using *SMA-TK* and *KPC* GEMM mice, both male and female mice were used. Animal sex was not considered in the study design. Tumors did not exceed the maximum tumor size of 1,500 mm^3^ permitted by the University of Massachusetts Chan Medical School IACUC. Though

### Pancreas transplant tumor models

5x10^4^ *KPC1*, 2.5x10^5^ *KPC2*, 5x10^4^ *KP1*, or 1x10^5^ *KP2* cells were resuspended in 25 μl of Matrigel (Matrigel, BD) diluted 1:1 with cold PBS and transplanted into the pancreas of 8-12 week old C57BL/6 female mice. Following anesthetization using 2-3% isoflurane, an incision was made in the left abdominal side and the cell suspension was injected into the tail region of the pancreas using a Hamilton Syringe. Successful injection was verified by the appearance of a fluid bubble without signs of intraperitoneal leakage. The abdominal wall was sutured with an absorbable Vicryl suture (Ethicon), and the skin was closed with wound clips (CellPoint Scientific Inc.). Mice were monitored for tumor development by ultrasound imaging, and randomized into treatment groups 1-week post-transplantation based on tumor volume. Upon sacrifice pancreas tumor tissue was allocated for 10% formalin fixation, OCT frozen blocks, flow cytometry analysis, and FACS sorting for downstream RNA-seq analysis.

### Lung transplant tumor models

5x10^5^ *KPC1*, 5x10^5^ *KPC2*, 4x10^4^ *KP1*, or 2.5x10^5^ *KP2* cells were resuspended in PBS and transplanted by tail vein injection into 8-12 week old C57BL/6 female mice. Mice were monitored for tumor development by bioluminescence imaging (BLI) on a Xenogen IVIS (Caliper Life Sciences) and randomized into various treatment cohorts 1-week post-transplantation. Upon sacrifice lung lobes were allocated for 10% formalin fixation (1 lobe), OCT frozen blocks (1 lobe), and flow cytometry analysis and FACS sorting (3 lobes).

### Liver transplant tumor models

2x10^5^ *KPC1* or *KP1* cells were resuspended in 25 μl of Matrigel (Matrigel, BD) diluted 1:1 with cold PBS and transplanted directly into the liver of 8-12 week old C57BL/6 female mice. Mice were monitored for tumor development by BLI on a Xenogen IVIS (Caliper Life Sciences) and randomized into various treatment cohorts 1-week post-transplantation. Upon sacrifice liver tumor tissue was allocated for 10% formalin fixation, OCT frozen blocks, and flow cytometry analysis.

### *KPC* GEMM model

*Trp53^fl/wt^, Kras^LSL-G12D/wt^* and *P48-Cre* strains on a C57BL/6 background were interbred to obtain *P48-Cre; Kras^LSL-G12D/wt^; Trp53^fl/wt^* (*KPC*) GEMM mice. Mice were monitored for tumor development by ultrasound imaging, and enrolled and randomized into treatment groups once tumors reached ~50 mm^3^ in volume. 23 male and 25 female mice were used for experiments. Upon sacrifice pancreas tumor tissue was allocated for 10% formalin fixation and OCT frozen blocks.

### Preclinical drug studies

Mice were treated with vehicle, trametinib (1 mg/kg body weight), palbociclib (100 mg/kg body weight) and/or tazemetostat (125 mg/kg (low) or 400 mg/kg (high) body weight) *per os* for 4 consecutive days followed by 3 days off treatment. For NK and T cell depletion, mice were injected intraperitoneally (IP) with an αNK1.1 (250 μg; PK136, BioXcell), αCD8 (200 μg; 2.43, BioXcell) or αCD4 (200 μg; GK1.5, BioXcell) antibody twice per week. Depletion of NK, CD4^+^, and CD8^+^ T cells was confirmed by flow cytometric analysis. For neutralization of chemokine signaling, mice were injected IP with an αCCL2 (200 μg; 2H5, BioXcell) or αCXCR3 (200 μg; CXCR3-173, BioXcell) antibody twice per week. No obvious toxicities were observed in treated animals. Ultrasound imaging was repeated every 2 weeks during treatment to assess changes in PDAC tumor burden.

### SMA depletion *in vivo*

5x10^4^ *KPC1* PDAC cells were transplanted orthotopically into 8-16 week old *SMA-TK* male (27) and female (18) mice. Mice were monitored for tumor development by ultrasound imaging, and randomized into treatment groups 1-week post-transplantation based on tumor volume. Mice were treated with vehicle or trametinib (1 mg/kg body weight) and palbociclib (100 mg/kg body weight) (T/P) *per os* for 4 consecutive days followed by 3 days off treatment, and GCV (50 mg/kg body weight) daily by IP injection. SMA depletion following GCV administration was confirmed by IHC analysis. Ultrasound imaging was repeated every 2 weeks during treatment to assess changes in PDAC tumor burden.

### Ultrasound Imaging

High-contrast ultrasound imaging was performed on a Vevo 3100 System with a MS250 13- to 24-MHz scanhead (VisualSonics) to stage and quantify PDAC tumor burden. Tumor volume was analyzed using Vevo 3100 software, version 5.50.

### Bioluminescence imaging

Bioluminescence imaging (BLI) was used to track *KPC1* PDAC and *KP1* LUAD tumor cells expressing a luciferase-GFP reporter following tail vein injection to stage and quantify lung tumor burden. Mice were injected IP with luciferin (5 mg/mouse; Gold Technologies) and then imaged on a Xenogen IVIS Spectrum imager (PerkinElmer) 10-15 minutes later for 60 seconds. Quantification of luciferase signaling in the thoracic region was analyzed using Living Image software, version 7.4.3 (Caliper Life Sciences).

### Flow cytometry

For analysis of MHC-I expression in cell lines cultured *in vitro*, cells were treated for 8 days with vehicle (DMSO), combined trametinib (25 nM) and palbociclib (500 nM), and/or tazemetostat (5 μM) and then trypsinized, resuspended in PBS supplemented with 2% FBS, and stained with an H-2k^b^ antibody (AF6-88.5.5.3, eBioscience; 1:200) for 30 minutes on ice. Flow cytometry was performed on a BD LSR II, and data were analyzed using FlowJo, version 10.8.1 (TreeStar).

For *in vivo* sample preparation, lungs were isolated, flushed with PBS, and allocated for 10% formalin fixation (1 lobe), OCT frozen blocks (1 lobe), and FACS (3 lobes) following 2-week treatment. Pancreatic tumor tissue was isolated from the spleen and normal tissue and allocated for 10% formalin fixation, OCT frozen blocks, and FACS following 2-week treatment. Liver tumors were isolated from liver lobes and allocated for 10% formalin fixation, OCT frozen blocks, and FACS following 2-week treatment. To prepare single cell suspensions for flow cytometry analysis, lung, pancreas, or liver tissue was minced with scissors into small pieces and placed in 5ml of collagenase buffer (1x HBSS w/ calcium and magnesium (Gibco), 1 mg/ml Collagenase A (Roche) for LUAD tumors or Collagenase V (Sigma-Aldrich) for PDAC tumors, and 0.1 mg/ml DNase I) in C tubes and then processed using program 37C_m_LDK_1 (for LUAD tumors) or 37C_m_TDK1_1 (for PDAC tumors) on a gentleMACS Octo dissociator with heaters (Miltenyi Biotec). Spleens were placed in 3 ml of PBS supplemented with 2% FBS in C tubes and dissociated using program m_spleen_01 on a gentleMACS Octo dissociator with heaters (Miltenyi Biotec). Dissociated tissue was passaged through a 70 μm cell strainer and centrifuged at 500 x g for 5 minutes. Red blood cells were lysed with ACK lysis buffer (Quality Biological) for 5 minutes, and samples were centrifuged and resuspended in PBS supplemented with 2% FBS. Samples were blocked with anti-CD16/32 (FC block, BD Pharmigen) for 20 minutes and then incubated with the following antibodies for 30 minutes on ice: CD45 AF700 (30-F11; 1:320), NK1.1 BV605 (PK136; 1:200), CD3 BV650 (17A2; 1:300), CD8 PE-Cy7 (53-6.7; 1:400), CD4 PE-Cy5 (GK1.5; 1:200), CD69 APC-Cy7 (H1.2F3; 1:200), Sca-1 PerCP-Cy5.5 (D7; 1:100), F4/80 APC (BM8; 1:200) (Biolegend); and CD11b (M1/70; 1:1,280) (BD Biosciences). NK cells were gated from the CD45^+^CD3^−^NK1.1^+^ population. DAPI was used to distinguish live/dead cells, and tumor cells were gated as GFP^+^. Flow cytometry was performed on an BD LSRFortessa or LSR II. Data was collected using BD FACSDiva Software, version 8.0 and analyzed using FlowJo, version 10.8.1 (TreeStar).

For analysis of Granzyme B (GZMB) expression in NK and T cells, single cell suspensions from tumor tissue were resuspended in RPMI media supplemented with 10% FBS and 100 IU/ml P/S and incubated for 4 hours with PMA (20 ng/ml, Sigma-Aldrich), Ionomycin (1 μg/ml, STEMCELL technologies), and monensin (2 μM, Biolegend) in a humidified incubator at 37°C with 5% CO_2_. Cell surface staining was first performed with CD45 AF700 (30-F11; 1:320), NK1.1 BV605 (PK136; 1:200), CD3 BV650 (17A2; 1:300), CD8 APC-Cy7 (53-6.7; 1:200), and CD4 PE-Cy5 (GK1.5; 1:200) (Biolegend). Intracellular staining was then performed using the Foxp3/transcription factor staining buffer set (eBioscience), where cells were fixed, permeabilized, and then stained with a GZMB antibody (GB11, Biolegend; 1:100). GZMB expression was evaluated by gating on CD3^−^NK1.1^+^ NK cells and CD3^+^CD8^+^ T cells on an BD LSR II flow cytometer as described above.

### NK and T cell degranulation assays

Mice were injected intravenously (i.v.) with 250 μl of a solution containing 25 μg anti-CD107a PE (ID4B, Biolegend) and 10 μg monensin (Biolegend) in PBS 4 hours before mice were euthanized. Tumor tissue was then isolated, dissociated into single cell suspensions, stained with cell surface antibodies, and analyzed by flow cytometry as described above.

### Immunohistochemistry (IHC)

Tissues were fixed overnight in 10% formalin, embedded in paraffin, and cut into 5 μm sections. Haematoxylin and eosin (H&E), Masson’s trichrome, and immunohistochemical staining were performed using standard protocols. Sections were de-paraffinized, rehydrated, and boiled in a pressure cooker for 20 minutes in 10 mM citrate buffer (pH 6.0) for antigen retrieval. Antibodies were incubated overnight at 4°C. The following primary antibodies were used: EZH2 (5246; 1:100), H3K27me3 (9733; 1:200), Cleaved Caspase-3 (CC3, 9664; 1:200) (Cell Signaling); Ki67 (AB16667; 1:100), CD3 (AB5690; 1:200), GZMB (AB4059; 1:100), CD31 (AB28364; 1:50), α-smooth muscle actin (αSMA, AB5694; 1:200) (Abcam); pRB^S807/S811^ (Sc-16670, Santa Cruz; 1:100); NKp46 (AF2225; 1:100), CXCL10 (AF466; 1:100) (R&D Systems); and CCL2 (MA5-17040, Invitrogen; 1:200). Prediluted HRP-conjugated secondary antibodies (Vectastain Elite ABC-HRP Kits: Rabbit, PK-6101; Mouse, PK-6102; Goat, PK-6105) were applied for 30 minutes and visualized with DAB (Vector Laboratories; SK-4100).

For quantification of proliferating pRb^+^ and Ki67^+^ cells, CC3^+^ dead/dying cells, CD31^+^ blood vessels, SMA^+^ fibroblasts, and NKp46^+^, CD3^+^, and GZMB^+^ immune cells, 5-10 high power 20x fields per section were counted and averaged using ImageJ software. The percentage of total lung area covered in tumor was quantified from H&E stained sections using ImageJ software to assess tumor burden in the lung in some experiments.

For H-score quantification of H3K27me3, EZH2, CCL2, and CXCL10 IHC staining intensity, tissue sections were first imaged using a TissueGnostics TissueFAXS SL slide scanning microscope with PixeLINK PL-D674CU-CYL-07451 / 674002030 camera and a Zeiss 20x 0.5NA air objective. Images were analyzed using StrataQuest image analysis software, version 7.1.1.129. Regions of interest containing tumor areas for analysis were selected manually. Image processing was done using a custom designed StrataQuest pipeline that performed color separation, nuclei identification, and channel intensity quantification in a blinded manner. Cutoffs were manually set for negative, low, medium, and high cells and the same values were applied to all images. H-score was calculated as previously described^78^.

### Immunofluorescence

Tissue sections were prepared for immunofluorescence staining using standard protocols as described above for IHC. The following primary antibodies were used: H3K27me3 (9733; 1:400) and GFP (2956; 1:200) (Cell Signaling); p21 (556431; 1:200) (BD Biosciences); and GFP (AB6673; 1:250) (Abcam). Secondary Alexa Fluor 488, 594, or 647 dye-conjugated antibodies (Invitrogen; 1:300) were applied for 1 hour at room temperature. Fluorescence antibody-labeled slides were mounted with Prolong Gold Antifade mountant (Prolong Molecular Probes; P36934) after counterstaining with DAPI. Analysis of the percentage of GFP^+^ tumor cells expressing p21 or H3K27me3 was performed using QuPath software, version 0.4.1.

### High throughput RNA-sequencing (RNA-seq)

For RNA-seq analysis of PIP, PIL, LIL, and LIP tumor samples, GFP^+^ tumor cells were FACS sorted on a FACSAria (BD Biosciences) from the lungs or pancreas of tumor-bearing mice following 2-week treatment with vehicle or combined trametinib (1 mg/kg body weight) and palbociclib (100 mg/kg). Total RNA was extracted from tumor cells using the RNeasy Mini Kit (Qiagen). Purified polyA mRNA was subsequently fragmented, and first and second strand cDNA synthesis performed using standard Illumina mRNA TruSeq library preparation protocols. Double stranded cDNA was subsequently processed for TruSeq dual-index Illumina library generation. For sequencing, pooled multiplexed libraries were run on a HiSeq 2500 machine on RAPID mode. Approximately 10 million 76bp single-end reads were retrieved per replicate condition. RNA-Seq data was analyzed by removing adaptor sequences using Trimmomatic, version 0.36^79^, aligning sequencing data to GRCm38 (Ensembl, version 101) with STAR, version 2.5.3a^80^, and genome wide transcript counting using featureCounts, version 1.6.3^81^ to generate a RPKM matrix of transcript counts. Genes were identified as differentially expressed using R package DESeq2, version 1.28.1 with a cutoff of absolute log_2_FoldChange ≥ 1 and adjusted p-value <0.05 between experimental conditions^82^. Heatmaps were generated using pheatmap, version 1.0.12. Over-representation analysis of differentially expressed genes (DEGs) against KEGG^83^ Pathways was performed using clusterProfiler, version 4.0.5^84^.

For RNA-seq analysis of *KPC1 shRen* and *shEzh2* PDAC tumors transplanted into C57BL/6 mice and parental *KPC1* PDAC tumors transplanted into *SMA-TK* mice, GFP^+^ tumor cells were FACS sorted on a FACSAria (BD Biosciences) from the pancreas of tumor-bearing mice following 2-week treatment with vehicle, combined trametinib (1 mg/kg body weight) and palbociclib (100 mg/kg), and/or GCV (50 mg/kg body weight). Total RNA was extracted from tumor cells using the RNeasy Mini Kit (Qiagen). Library preparation and sequencing on a NovaSeq 6000 was performed by Novogene. Approximately 30 million 150bp paired-end reads were retrieved per replicate condition. Quality of raw sequencing data was checked using FastQC, version 0.11.5 (https://www.bioinformatics.babraham.ac.uk/projects/fastqc/) to assure data quality. Paired-end reads were aligned to the mouse reference genome GRCm38 (Ensembl, version 101) using STAR, version 2.5.3a^80^. A gene-by-sample count matrix was generated using featureCounts, version 1.6.2^81^. All downstream statistical analyses were done using the R programming language, version 4.1.0^85^. Briefly, entries of genes with extremely low expression were first removed from the gene-by-sample count matrix. Differential gene analysis was performed using DESeq2, version 1.32.0^82^ in consideration of surrogate variables for hidden variations, which were identified using svaseq, version 3.40.0^86^. Genes with absolute values of log_2_ (fold change) greater than one and *p*-values less than 0.05, which were corrected for multiple comparisons using the Benjamini-Hochberg procedure^87^, were considered as significantly DEGs. Over-representation analysis of DEGs against KEGG^83^ and REACTOME^88^ Pathways was performed using clusterProfiler, version 4.0.5^84^. For heatmap visualization of selected genes and pathways, samples were z-score normalized and plotted using pheatmap, version 1.0.12.

To assess expression of SASP genes in human PDAC and LUAD cell lines treated with vehicle (DMSO), trametinib (25 nM), and/or palbociclib (500 nM) for 8 days in culture, we interrogated a previously published RNA-seq dataset under the GEO accession number GSE110397^19^. Graphs display normalized RPKM expression values.

### Gene Set Enrichment Analysis (GSEA)

GSEA was performed using the GSEAPreranked tool for conducting gene set enrichment analysis of data derived from RNA-seq experiments (version 2.07) against EZH2 target gene sets and published senescence signatures^40^. The metric scores were calculated using the sign of the fold change multiplied by the inverse of the p-value.

### Transcription factor enrichment analysis

Transcription factor enrichment analysis was performed on DEGs using gene set libraries from Enrichr, version 3.0^89^. Significance of the tests was assessed using combined score, described as c = log(p) * z, where c is the combined score, p is Fisher exact test p-value, and z is z-score for deviation from expected rank.

### CUT&Tag analysis

CUT&Tag was performed largely as previously described^45^. For *in vivo* CUT&tag analysis of PIP tumor samples, GFP^+^ tumor cells were FACS sorted on a FACSAria (BD Biosciences) from the pancreas of tumor-bearing mice following 2-week treatment with vehicle or combined trametinib (1 mg/kg body weight) and palbociclib (100 mg/kg). For *in vitro* CUT&Tag analysis, *KPC1* PDAC cell lines were treated for 8 days with vehicle or combined trametinib (25 nM) and palbociclib (500nM). 100,000 cells per condition were then resuspended in wash buffer (20 mM HEPES pH 7.5; 150 mM NaCl; 0.5 mM Spermidine; 1x Protease inhibitor cocktail). 10 μl of activated Concanavalin A coated magnetic beads (Polysciences) were added per sample and incubated at room temperature (RT) for 15 min. Bead-bound cells were resuspended in 100 μl Dig-wash Buffer (20 mM HEPES pH 7.5; 150 mM NaCl; 0.5 mM Spermidine; 0.05% Digitonin; 1x Protease inhibitor cocktail) containing 2 mM EDTA and 1 μl of H3K27me3 antibody (ThermoFisher, MA5-11198; 1:100). The mixture was incubated overnight at 4 °C for antibodies to bind. After pulling beads to the side of the tube using a magnetic rack and removal of unbound primary antibody, beads were resuspended in 100 μl Dig-wash Buffer containing 1 μl of Guinea Pig anti-Rabbit antibody (Antibodies-Online, ABIN101961; 1:100) and incubated for 30 mins at RT. Cells were washed 3 times with Dig-wash and then incubated with a 1:50 dilution of pA-Tn5 adapter complex in Dig-med (0.05% Digitonin, 20 mM HEPES, pH 7.5, 300 mM NaCl, 0.5 mM Spermidine, 1x Protease inhibitor cocktail) at RT for 1 hr. Cells were washed thrice in Dig-med Buffer and then resuspended in 300 μl Dig-med Buffer containing 10 mM MgCl_2_ and incubated at 37 °C for 1 hr to activate tagmentation. To stop tagmentation, 10 μl of 0.5 M EDTA, 3 μl of 10% SDS and 1 μl of 20 mg/ml Proteinase K was added to each tube, which were incubated at 55 °C for 1 hr. DNA was extracted by performing one phenol:chloroform extraction followed by ethanol precipitation. The DNA pellet was resuspended in 22 μl of 10 mM Tris pH 8.

CUT&Tag libraries were amplified by mixing 21 μl of tagmented DNA with 2μl each of (10 μM) barcoded i5 and i7 primers^90^, using a different combination for each sample. 25 μl NEBNext HiFi 2x PCR Master mix (NEB) was added to each, and PCR was performed using the following cycling conditions: 72 °C for 5 min (gap filling); 98 °C for 30 s; 17 cycles of 98 °C for 10 s and 63 °C for 30 s; final extension at 72 °C for 1 min and holding at 4 °C. 1.1x volumes of Ampure XP beads (Beckman Coulter) were incubated with libraries for 10 min at RT to clean up the PCR reaction. Bead bound DNA was purified by washing twice with 80% ethanol and eluting in 20 μl 10 mM Tris pH 8.0.

The libraries were quantified by Qubit and paired-end sequencing was performed on an Illumina NextSeq 500 (38 bases for reads 1 and 2 and 8 base indexing on both ends) or on a NovaSeq 6000 (150bp paired-end reads). At least 3 million reads were retrieved per replicate condition. Paired-end reads were aligned to the mouse reference genome GRCm38 (Ensembl, version 101) using bwa mem^91^ after quality assurance with FastQC, version 0.11.5. Alignment files in the SAM format were first sorted by coordinates and converted into the BAM format using SAMtools, version 1.9^92^. Subsequently, PCR duplicates were removed from the BAM files using “MarkDuplicates” command of the Picard tools, version 2.9.0 (https://broadinstitute.github.io/picard/). The resulting BAM files were name sorted using SAMtools again. Peaks per condition were called using Genrich, version 0.6 (https://github.com/jsh58/Genrich) with name-sorted, de-duplicated BAM files of all biological replicates for a given condition as input and a q-value cutoff of 0.05. Given that H3K27me3 modification are widespread across inactive gene regions, peaks with sizes less than 1 kb were filtered out. Genome coverage per sample was calculated by converting sorted, de-duplicated BAM into bigwig files using bamCoverage command (deeptools, version 3.0.2)^93^. Median genome coverage per condition was calculated using wiggletools^94^, version 1.2 with all bigWig files for the given condition as input. A matrix containing the median signal of all conditions for the consensus set of H3K27me3 CUT&Tag peak regions and for transcripts of 87 SASP genes (see Supplementary Table 47) ± 3kb flanking the peak regions was calculated from the median genome coverage of each condition using computeMatrix reference-point command and visualized using plotProfile command^93^. Consensus peaks-by-sample count matrix were determined using DiffBind, version 3.4.11^95^. Differential peak analysis was conducted using DEseq2, version 1.32.0^82^ with hidden variations adjusted for using svaseq, version 3.40.0^86^. Peaks with absolute values of log_2_ (shrunken fold change) greater than one and *p*-values less than 0.05, which were corrected for multiple testing using the Benjamini-Hochberg procedure^87^, were considered as significantly differential peaks. Track views were generated using the Integrative Genomics Viewer (IGV), version 2.8.9^96^.

### Pearson’s correlation analysis

Gene expression data from 145 primary PDAC tumors (GSE71729)^55^ was downloaded with GEOquery2 package, version 2.62.2. Correlation analysis between PRC2^97^ and our custom EZH2 repressed gene sets (generated from DEGs upregulated in *shEzh2* compared to *shRen* PDAC tumor cells from RNA-seq analysis in Fig. 6), inflammatory response, NK cell^98^, and CD8^+^ T cell^99^ gene sets (see Supplementary Table 47), and *CCL2*, *CXCL9*, and *CXCL10* expression was performed using ggpubr package, version 0.4.0. Pearson’s correlation coefficient (R) values are displayed.

### Human PDAC specimens

PDAC patient samples were derived retrospectively from surgical candidates undergoing Whipple or distal pancreatectomy procedures at UMass Memorial Hospital between 09/18/2017 and 05/05/2022 consented under the IRB approved protocol no. H-4721. Samples from 20 male and 14 female patients (based on self-reporting) were used for analysis, though sex and/or gender was not considered in the study design. De-identified FFPE tumor specimens were cut into 5 μm sections and IHC performed using a DAKO Auto Stainer Plus according to manufacturers’ protocols to stain for human EZH2 (5246, Cell Signaling; 1:100), NKp46 (AF1850, R&D Systems; 1:50), and CD8 (C8/144B, Dako, 1:200). Prediluted HRP-conjugated secondary antibodies (Dako EnVision Duel-link System-HRP, K4061) were applied for 60 minutes and visualized with DAB (Dako, K3468). Scoring was performed in a blinded manner. EZH2 staining was scored as high (strong nuclear staining throughout tumor), intermediate (nuclear staining in some but not all tumor areas), or low (little to no positive staining in the tumor). NKp46^+^ NK cell numbers were scored as high (> 5 cells per 40x field), intermediate (2-4 cells per 40x field), or low (< 2 cells per 40x field). CD8^+^ T cell numbers were scored as high (> 10 cells per 40x field), intermediate (5-10 cells per 40x field), or low (< 5 cells per 40x field). Survival data from PDAC patients was also available through the IRB approved protocol no. H-4721.

### Statistics and Reproducibility

Statistical analyses were performed as described in the figure legend for each experiment. Data are expressed as mean ± SEM. No statistical methods were used to pre-determine sample sizes but our sample sizes are similar to those reported in previous publications^19,20^. The indicated sample size (*n*) represents biological replicates and measurements were taken from distinct samples. All samples that met proper experimental conditions were included in the analysis. No data were excluded from the analyzes, except: (1) some mice had to be excluded from flow cytometry analysis if no detectable tumor could be found by gross examination; (2) some mice had to be excluded from ultrasound tumor volume and IHC analysis if tumors were largely necrotic; and (3) some samples for RNA-seq and CUT&Tag analysis had to excluded due to poor library quality and/or low reads. All experiments were repeated independently 2-3 times with similar results. For in vivo experiments, mice were randomized based on tumor burden as assessed by ultrasound or IVIS imaging to achieve equal tumor volume between experimental groups. For in vitro experiments sample allocation was performed randomly. Scoring of IHC staining in mouse and human tumor samples was performed in a blinded manner. For other experiments, data collection and analysis were not performed blind to the conditions of the experiments. Statistical significance was determined by two-sided Student’s *t*-test, log-rank test, One-way ANOVA followed by Tukey’s multiple comparison test, hypergeometric test, Kolmogorov-Smirnov test, and Wald test followed by corrections for multiple comparisons using the Benjamini-Hochberg procedure with Prism 9 software (GraphPad) and R. Data distribution was assumed to be normal but this was not always formally tested. Significance was set at *P* < 0.05.

### Reporting Summary

Further information on research design is available in the Nature Research Reporting Summary linked to this article.

## Extended Data

## Supplementary Material

Unprocessed Gels

Reporting Summary

Supplementary Tables 1-49

Source Data

Editorial checklist

Supplementary Fig. 1

## Data Availability

RNA-seq and CUT&Tag data that support the findings of this study have been deposited in the Gene Expression Omnibus (GEO) under accession nos. GSE141684, GSE201495, and GSE203623. Datasets derived from this resource that support the findings of this study are available in Supplementary Tables 1–46. Gene expression data for human LUAD and PDAC cell lines treated with T/P were obtained under accession no. GSE110397. Gene expression data from 145 primary human PDAC specimens were obtained under accession no. GSE71729. Source data for Figs. 1–8 and Extended Data Figs. 1–9 have been provided as Source data files. All other data supporting the findings of this study are available from the corresponding author upon reasonable request.
