# Supplementary material for: EZH2 inhibition remodels the inflammatory senescence-associated secretory phenotype to potentiate pancreatic cancer immune surveillance
Source: Nat Cancer. Author manuscript; Available in PMC 2023 Sep 22. (PMC10516132; doi:10.1038/s43018-023-00553-8)

Fig. 4a (top)

β-actin

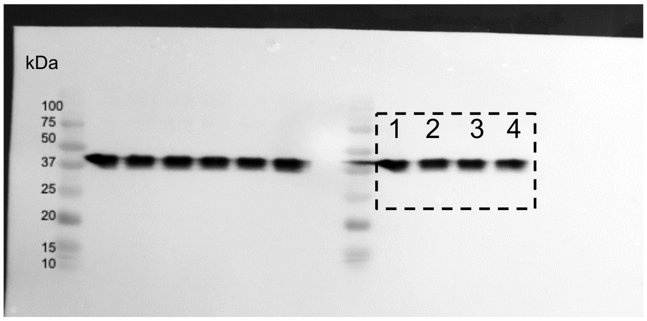

= used

EZH2

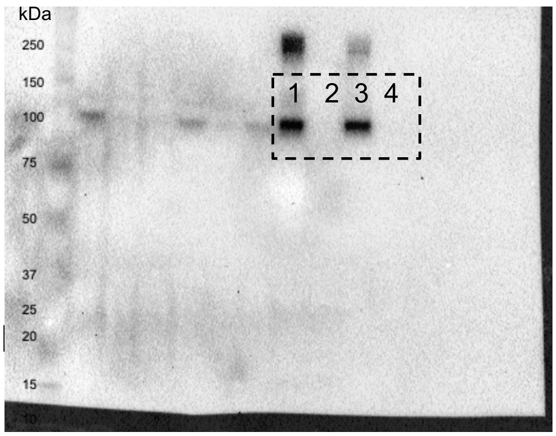

H3K27me3

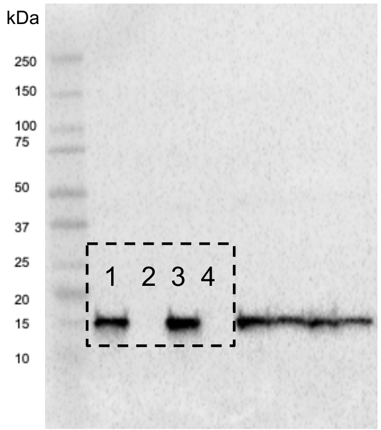

1. KPC1 shRen
2. KPC1 shEzh2
3. KPC2 shRen
4. KPC2 shEzh2

Fig. 4a (bottom)

β-actin

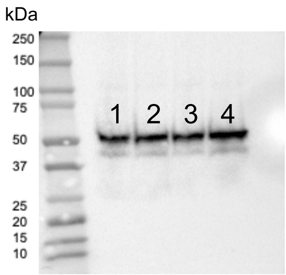

SUZ12

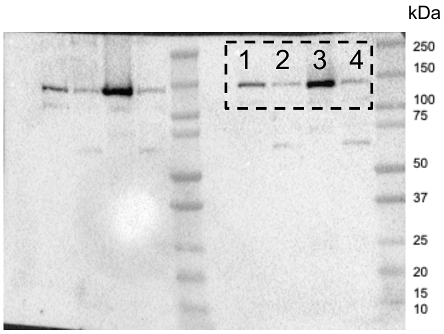

H3K27me3

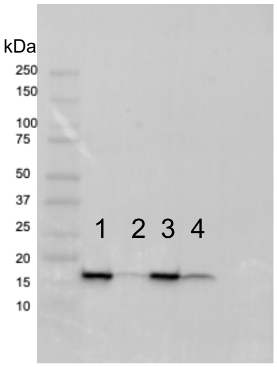

1. KPC1 shRen
2. KPC1 shSuz12
3. KPC2 shRen
4. KPC2 shSuz12

Fig. 4c

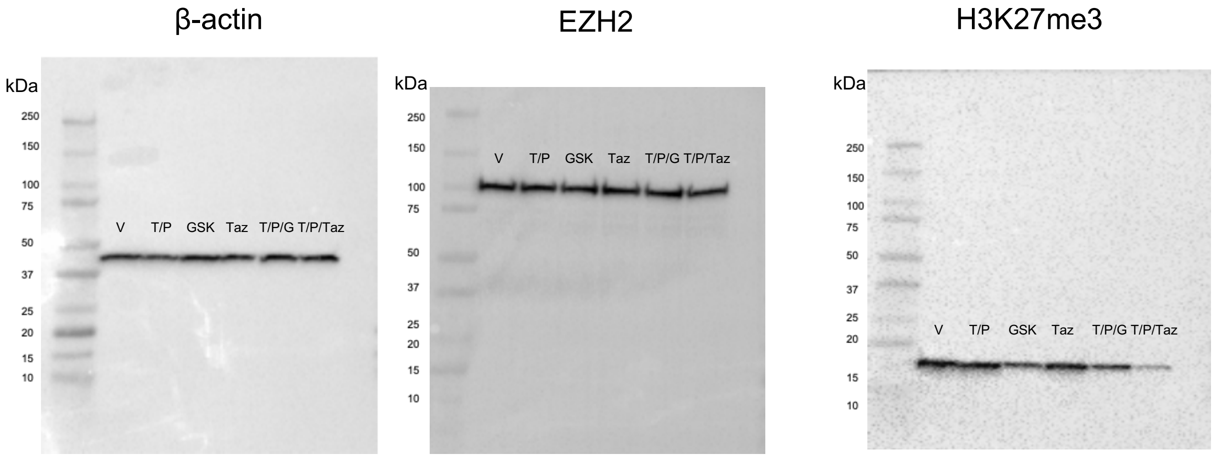
β-actin
EZH2
H3K27me3
kDa
250
150
100
75
50
37
25
20
15
10
V
T/P
GSK
Taz
T/P/G
T/P/Taz

Fig. 7a

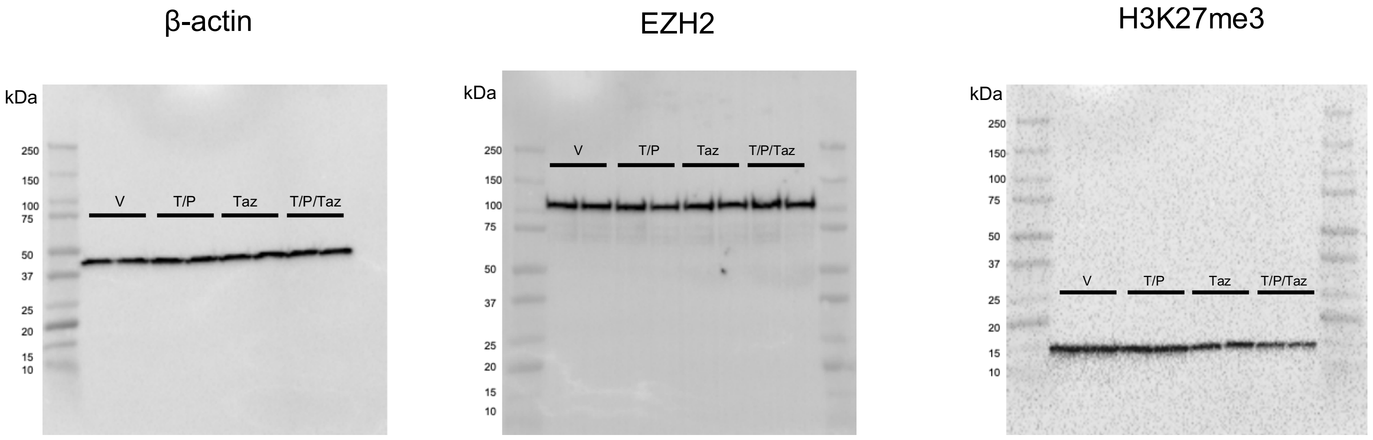
β-actin
EZH2
H3K27me3
kDa
250
150
100
75
50
37
25
20
15
10
V
T/P
Taz
T/P/Taz

Extended Data Fig. 7b

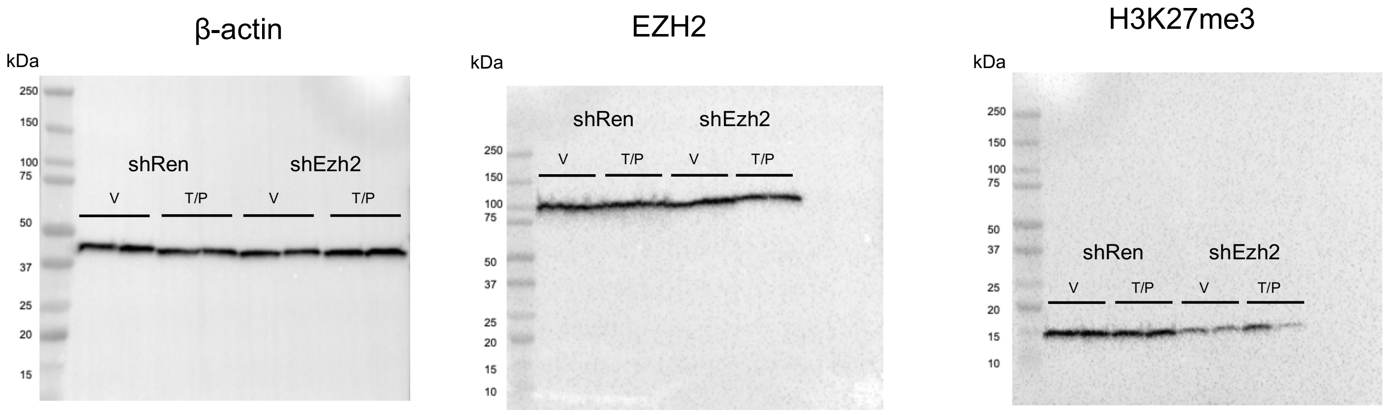

Supplement: Unprocessed Gels [file NIHMS1930095-supplement-Unprocessed_Gels.pdf]
