# Supplementary material for: EZH2 inhibition remodels the inflammatory senescence-associated secretory phenotype to potentiate pancreatic cancer immune surveillance
Source: Nat Cancer. Author manuscript; Available in PMC 2023 Sep 22. (PMC10516132; doi:10.1038/s43018-023-00553-8)

Supplementary Figure 1

A Gating Strategy for innate and adaptive immune cells and CD107a expression

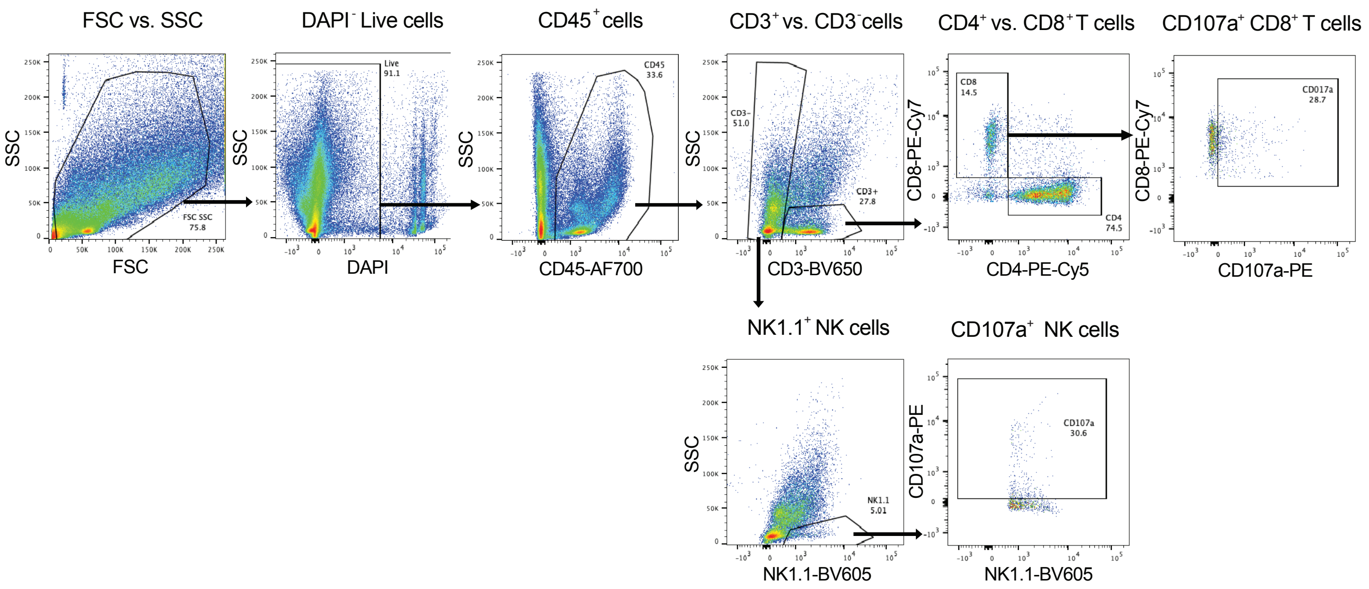

B Gating Strategy for innate and adaptive immune cells and Sca-1 and CD69 expression

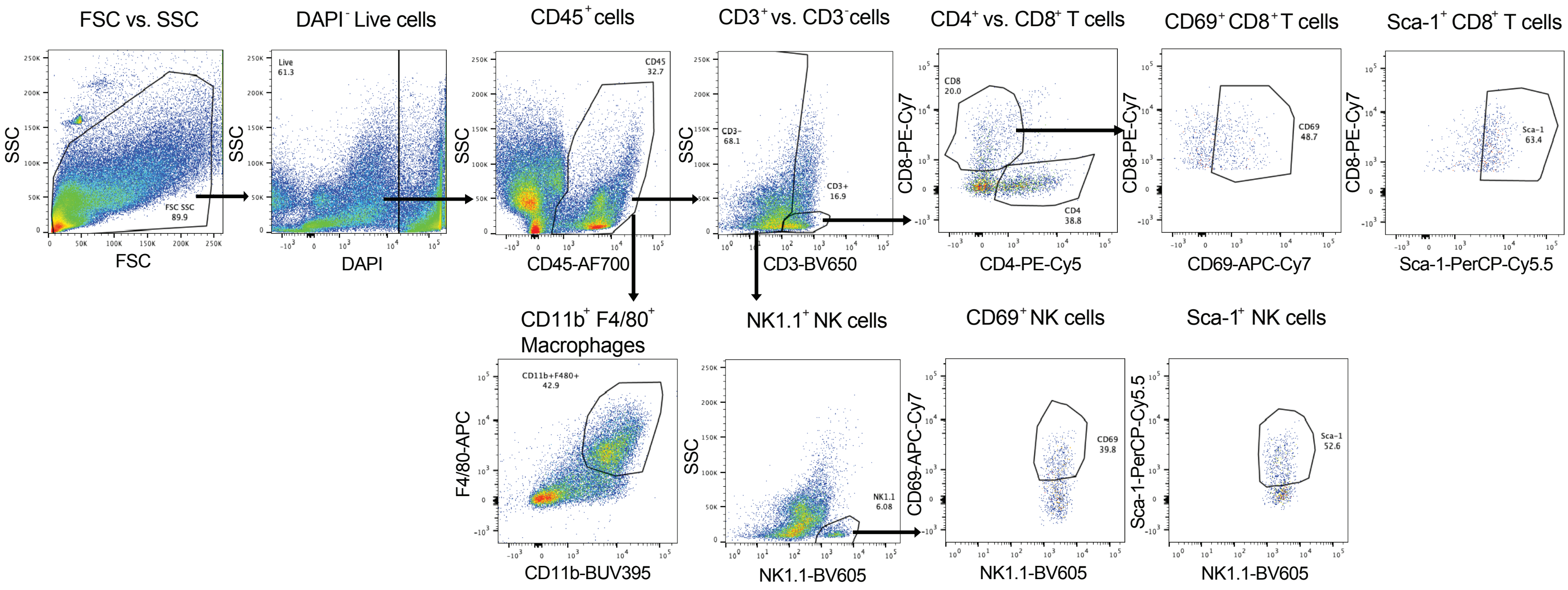

C Gating Strategy for GZMB expression following *in vitro* stimulation

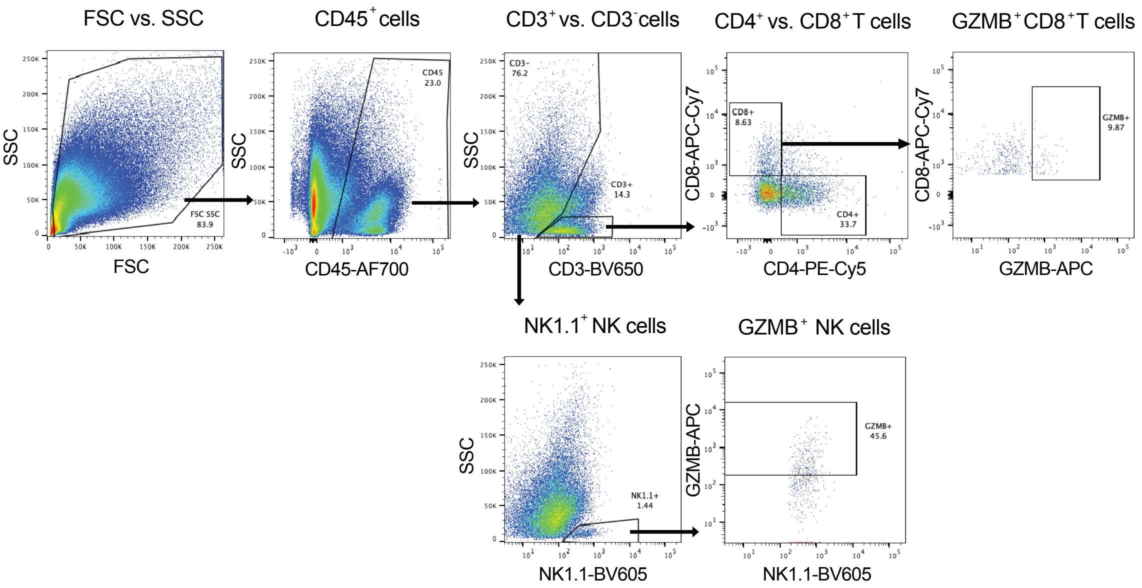

Supplement: Supplementary Fig. 1 [file NIHMS1930095-supplement-Supplementary_Fig__1.pdf]
